# Discovery of Novel Orally Active Anti-Inflammatory *N*-Phenylpyrazolyl-*N*-Glycinyl-Hydrazone Derivatives That Inhibit TNF-α Production

**DOI:** 10.1371/journal.pone.0046925

**Published:** 2012-10-08

**Authors:** Renata B. Lacerda, Leandro L. da Silva, Cleverton K. F. de Lima, Eduardo Miguez, Ana Luisa P. Miranda, Stefan A. Laufer, Eliezer J. Barreiro, Carlos A. M. Fraga

**Affiliations:** 1 Laboratório de Avaliação e Síntese de Substâncias Bioativas (LASSBio), Faculty of Pharmacy, Federal University of Rio de Janeiro, Rio de Janeiro, Rio de Janeiro, Brazil; 2 Programa de Pós-Graduação em Química, Chemistry Institute, Federal University of Rio de Janeiro, Rio de Janeiro, Rio de Janeiro, Brazil; 3 Programa de Pós-Graduação em Farmacologia e Química Medicinal, Institute of Biomedical Sciences, Federal University of Rio de Janeiro, Rio de Janeiro, Rio de Janeiro, Brazil; 4 Institute of Macromolecules Professora Eloisa Mano, Federal University of Rio de Janeiro, Rio de Janeiro, Rio de Janeiro, Brazil; 5 Department of Pharmaceutical and Medicinal Chemistry, Institute of Pharmacy, Eberhard Karls University of Tübingen, Tuebingen, Germany; Universidad Federal de Santa Catarina, Brazil

## Abstract

Herein, we describe the synthesis and pharmacological evaluation of novel *N*-phenylpyrazolyl-*N*-glycinyl-hydrazone derivatives that were designed as novel prototypes of p38 mitogen-activated protein kinase (MAPK) inhibitors. All of the novel synthesized compounds described in this study were evaluated for their in vitro capacity to inhibit tumor necrosis factor α (TNF-α production in cultured macrophages) and in vitro MAPK p38α inhibition. The two most active anti-TNF-α derivatives, (*E*)-2-(3-tert-butyl-1-phenyl-1*H*-pyrazol-5-ylamino)-*N*’-((4-(2-morpholinoethoxy)naphthalen-1-yl)methylene)acetohydrazide (4a) and (*E*)-2-(3-tert-butyl-1-phenyl-1*H*-pyrazol-5-ylamino)-N’-(4-chlorobenzylidene)acetohydrazide (4f), were evaluated to determine their *in vivo* anti-hyperalgesic profiles in carrageenan-induced thermal hypernociception model in rats. Both compounds showed anti-inflammatory and antinociceptive properties comparable to SB-203580 used as a standard drug, by oral route at a dose of 100 µmol/kg. This bioprofile is correlated with the ability of NAH derivatives (4a) and (4f) suppressing TNF-α levels *in vivo* by 57.3 and 55.8%, respectively.

## Introduction

The production of proinflammatory cytokines, *e.g*., TNF-α, IL-1β and IL-6, is a key factor in chronic inflammatory diseases, such as rheumatoid arthritis, Crohn’s disease, psoriasis and asthma [1], [2]. Moreover, evidence exists that supports the involvement of cytokines in other diseases, including cardiac heart failure, ischemic retinopathy [3] and the development of insulin resistance in diabetes [4]. Due to the role of cytokines in various inflammatory diseases, many pharmaceutical companies have made efforts to develop new orally active substances that can modulate the production of proinflammatory cytokines.

Tumor necrosis factor-alpha (TNF-α) is a pleiotropic cytokine that possesses proinflammatory and osmoregulator actions [5]. It is the major cytokine mediator of acute inflammation, it activates platelets, and it is also involved in the genesis of fever and anemia. TNF-α also mediates many inflammatory events in rheumatoid arthritis, including immune cell activation, proliferation, apoptosis and regulation of leukocyte movement [6], which has led to the development of strategies to block TNF-α-mediated effects. The currently available anti-TNF-α strategies involve either administration of anti-TNF-α antibodies or soluble TNF receptors to remove circulating TNF-α [7]. These inhibitors act by binding to TNF-α and preventing it from binding to its receptors on nearby cells, thus preventing the initiation of apoptosis or an inflammatory response [8].

Despite the approval of anti-TNF-α drugs, e.g., infliximab, etanercept and adalimumab, which demonstrated the effectiveness of therapeutic strategies based on the depletion of TNF-α, the appearance of side effects resulting from the debilitating actions of these drugs on the immune system highlights the necessity of identifying new alternative mechanisms to modulate the actions of pro-inflammatory cytokines [9], [10].

**Figure 1:**
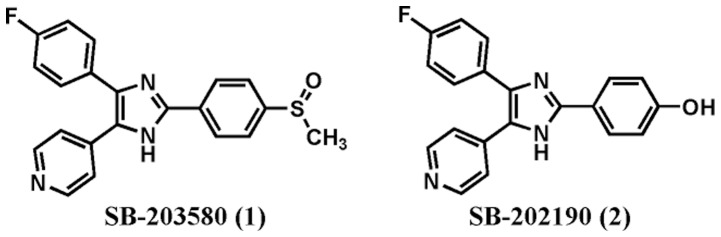
Pyridinyl-imidazole inhibitors of p38 MAPK.

One of the most promising targets involved in modulating the production of pro-inflammatory cytokines is the mitogen-activated protein kinase (MAPK) pathway, particularly p38 MAPK, a serine–threonine protein kinase that has been identified as a molecular target of the pyridinyl-imidazole derivatives SB-203580 (1) and SB-202190 (2) (Figure 1) [11], [12]. These terphenyl-heterocyclic derivatives, which have been widely used to study p38 MAPK function, competitively bind at the ATP-binding pocket of p38 MAPK and inhibit TNF-α and IL-1β production. Over the years, a large number of structurally diverse p38α and p38β MAPK inhibitors have been developed with both enhanced potency and specificity. Most of the p38 MAPK inhibitors are ATP competitors [13], but a new class of allosteric inhibitors has also been reported [14]. For example, BIRB-796 [15] (3) produces a conformational reorganization of the kinase that prevents ATP binding and activation.

**Figure 2:**
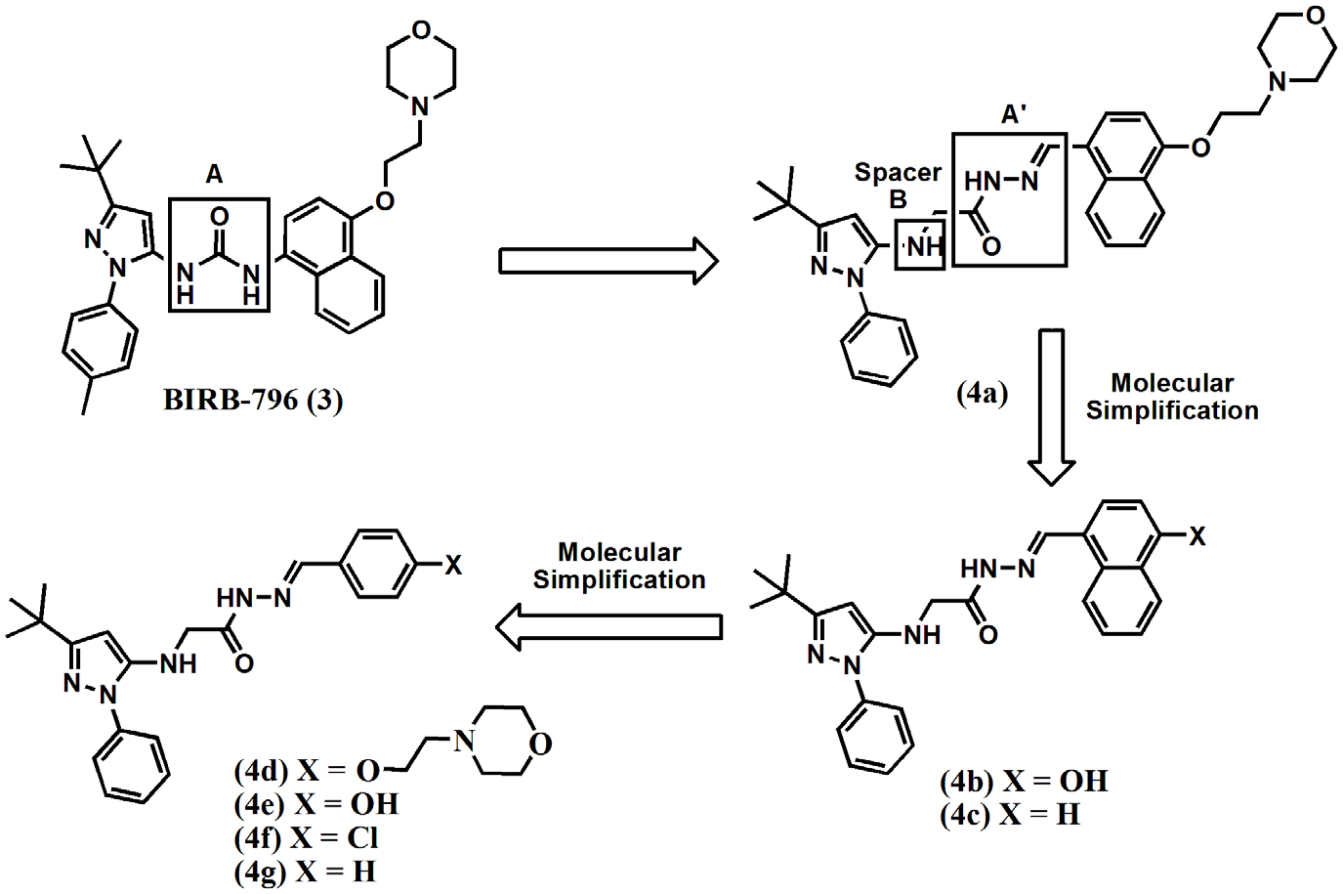
Design concept of novel *N*-phenylpyrazolyl-*N*-glycinyl-hydrazones derivatives 4a–g.

In this context, the present work describes the synthesis of novel *N*-phenylpyrazolyl-*N*-glycinyl-hydrazone derivatives 4a-g, which were designed as structural analogues of the p38 MAP kinase inhibitor BIRB-796 (3), and the investigation of their anti-cytokine and anti-inflammatory properties. For the proposed derivatives (4a–g), we investigated the replacement of the urea subunit of BIRB-796 (3) by a *N*-acylhydrazone unit [16] (A’, Figure 2), which was attached to the *N*-phenyl-pyrazole nucleus through an NHCH_2_ spacer (B, Figure 2).

Furthermore, we performed a series of molecular simplifications in the functionalized naphthyl framework attached to the imine unit of the NAH group of compound 4a to better understand the structure-activity relationships (Figure 2).

## Results and Discussion

**Figure 3:**
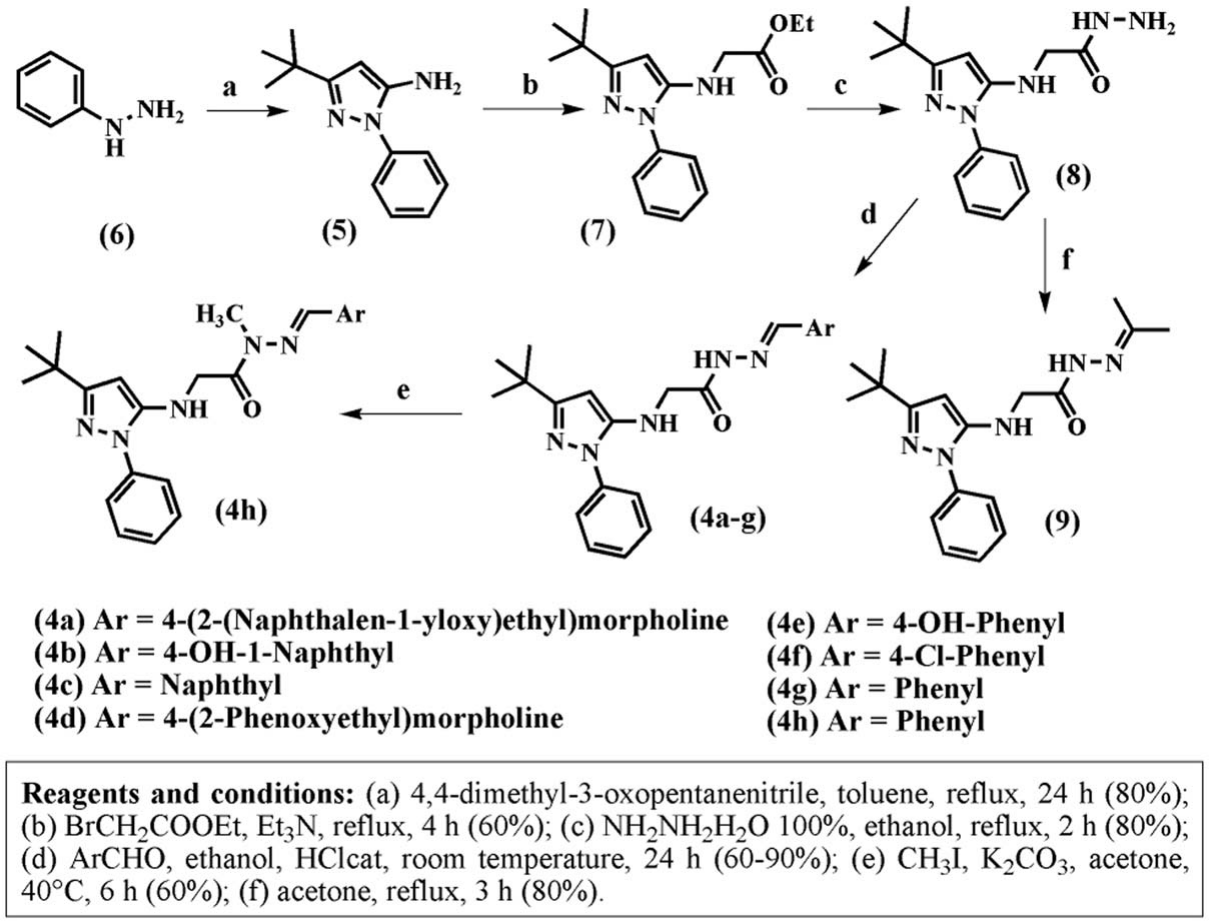
Synthesis of *N*-phenylpyrazolyl-*N*-glycinyl-hydrazone derivatives 4a–h.

The first step to obtain the *N*-phenylpyrazolyl-*N*-glycinyl-hydrazone derivatives 4a–g consisted of preparing the derivative 3-*tert*-butyl-1-phenyl-1H-pyrazol-5-amine (5) [15] from the condensation reaction between 4,4-dimethyl-3-oxopentanenitrile and phenylhydrazine (6) in refluxing toluene. The amino-pyrazole derivative 5 was subjected to alkylation with ethyl 2-bromoacetate in toluene and triethylamine under reflux, which gave rise to the corresponding amino ester 7 with a 60% yield. Next, the hydrazinolysis of the ester 7 with hydrazine hydrate in ethanol under reflux produced the corresponding hydrazide intermediate 8 with an 80% yield. The novel *N*-phenyl-pyrazolyl-*N*-glycinyl hydrazone derivatives 4a–g (Table 1) were then prepared in satisfactory yields through the acid catalyzed condensation of hydrazide 8 with aromatic aldehydes at room temperature (Figure 3).

**Table 1:** Yields and physical properties of *N*-phenyl-pyrazolyl-*N*-glycinyl-hydrazone derivatives 4a-g.

Compound	Molecular Formula[a]	MW	Yield (%)[b]	M.P.(oC)
4a	C_32_H_38_N_6_O_3_.(H_2_O)	572.70	70	180
4b	C_26_H_27_N_5_O_2_	441.52	70	270
4c	C_26_H_27_N_5_O	425.53	90	140
4d	C_28_H_36_N_6_O_3_	504.62	60	158
4e	C_22_H_25_N_5_O_2_	391.47	70	280
4f	C_22_H_24_ClN_5_O	409.91	70	193
4g	C_22_H_25_N_5_O	375.47	70	148

[a] The analytical results for C, H and N were within 0.4% of the calculated values.

[b] Yields obtained for the condensation step of hydrazide (8) with the corresponding aromatic aldehydes.

The structures of the *N*-phenyl-pyrazolyl-*N*-glycinyl-hydrazones 4a-g were completely characterized by common spectroscopic methods and the analytical results for C, H and N were within ±0.4% of the calculated values.

**Figure 4:**
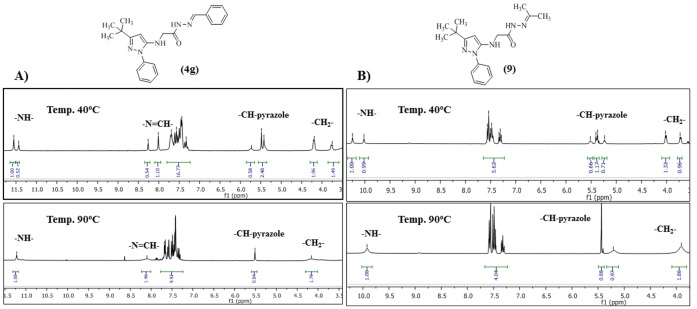
^1^H-NMR spectra of NAH derivatives 4g (A) and 9 (B) in DMSO-d_6_ at 40°C and 90°C.

**Figure 5:**
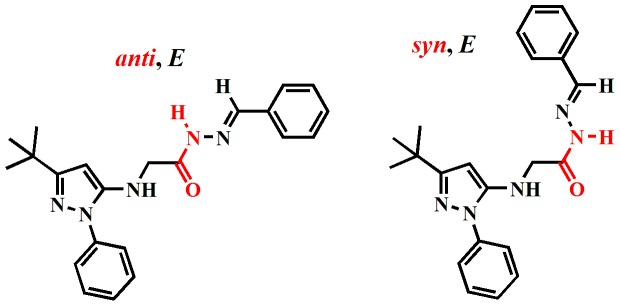
Probable conformational isomers of the NAH derivative 4 g.

According to the literature, *N*-acylhydrazones (NAHs) may exist as *Z*/*E* geometrical isomers about the C = N double bond and syn/anti amide conformers [17]. For most NAH derivatives described herein, the 1H-NMR spectra were recorded at room temperature, and they indicated the presence of two isomers, whereas only one species was detected by reversed-phase HPLC (Figure S22). In a study involving compound 4 g, the 1H-NMR spectrum in DMSO-d6 at 90°C showed that the two isomers were in rapid equilibrium (Figure 4A and Figure S13) [18]. Interestingly, complete coalescence of the signals was reached at 90°C, and the reversibility of the changes was verified, indicating the presence of conformational isomers (Figure 5). Moreover, the 1D NOESY showed spatial relationships of amide and imine hydrogens of compound 4 g that were compatible with the relative configuration (*E*) at the imine double bond (Figure S14, Figure S15 and Figure S23).

Another approach that was used to evaluate the presence of mixtures of conformers in our series of NAH derivatives 4a–g was based on the work of Wyrzykiewicz and Palla [17], [18]. A ^1^H-NMR spectrum of the compound 2-(3-*tert*-butyl-1-phenyl-1H-pyrazol-5-ylamino)-*N*’-(propan-2-ylidene)acetohydrazide (9), which was obtained by a reaction of the previously obtained hydrazide 8 with acetone (Figure 3), was performed because compound 9 cannot exist as *E*/*Z* geometrical isomers about the imine double bond. Nevertheless, the ^1^H-NMR spectrum of compound 9 displayed duplicate signals for amide, methylene and pyrazole hydrogens, which completely coalesced at 90°C (Figure 4B and Figure S18).

**Figure 6:**
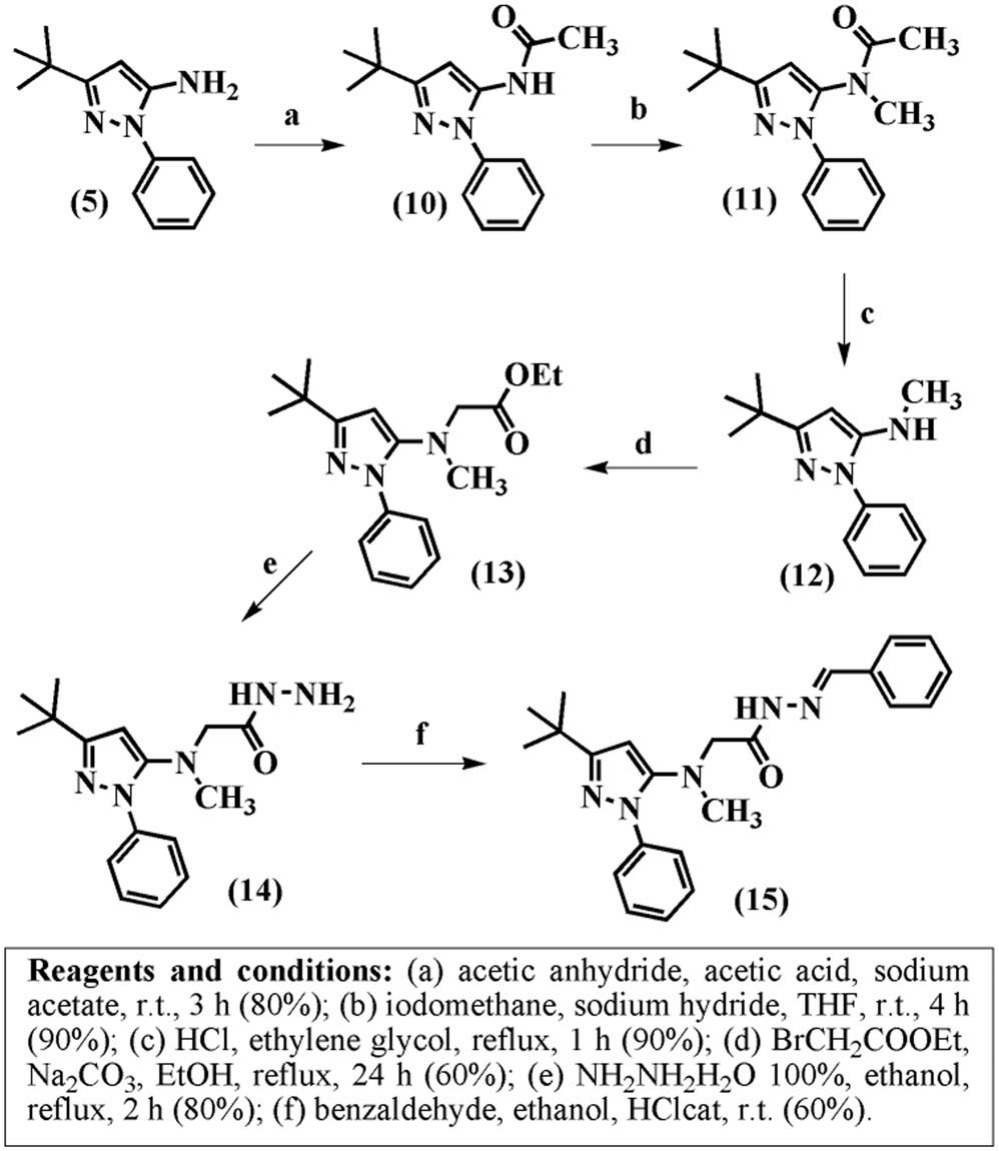
Synthesis of compound 15.

To evaluate whether the amino spacer exerts some influence on the stabilization of the conformational isomers in solution, we inserted a methyl group into the amino spacer, as described in Figure 6. The protection of the primary amine group [19] of compound 5 by treatment with acetic anhydride in acetic acid and sodium acetate resulted in the acetamide compound 10 with an 80% yield. Subsequent *N*-methylation was performed by deprotonation of compound 10 with NaH in THF followed by the addition of CH_3_I, which resulted in a 90% yield of *N*-methylacetamide 11. The next step consisted of the removal of the protecting acetyl group to obtain the 3-*tert*-butyl-*N*-methyl-1-phenyl-1H-pyrazol-5-amine (12), which showed a 90% yield. The alkylation of the monomethylamine derivative 12 with ethyl 2-bromoacetate in ethanol and sodium carbonate under reflux provided the corresponding ethyl ester 13 with a 60% yield. Hydrazinolysis of the ester 13 followed by condensation of the corresponding hydrazide 14 with benzaldehyde under acid catalysis resulted in the desired *N*-acylhydrazone derivative 15 with a 60% yield.

The ^1^H-NMR spectrum of the *N*-methyl derivative 15 showed the same pattern of duplicity that was observed for the other synthesized *N*-acylhydrazones 4a-g. We were also able to observe conformational isomers of the amide unit of compound 15, suggesting that the amino spacer does not participate as a hydrogen bond donor in the stabilization of the conformational isomers in solution (Figure S20 and Figure S21).

We also performed the chemoselective *N*-alkylation of the *N*-acylhydrazone derivative 4g (Figure 3) to investigate the influence of an alkyl group on the observation of conformational isomers in solution.

The pattern of duplication observed in the ^1^H NMR spectrum of the *N*-acylhydrazone derivative 4g disappeared after methylation of the NAH framework, *i.e*., we did not observe conformational isomers for the corresponding *N*-methyl *N*-acylhydrazone derivative 4h. These results suggest that the insertion of the methyl group at the amide nitrogen leads to a steric or electronic effect that does not allow the distinction of conformational isomers in solution by ^1^H-NMR (Figure S19), as previously reported by Kummerle and co-workers [20].

We initially investigated the capacity of our *N*-acylhydrazone derivatives 4a–g to inhibit TNF-α production *in vitro* [21]. The p38 MAPK inhibitor SB-203580 (1) was chosen as a standard. As depicted in Table 2, six NAH compounds 4a, 4b, 4c, 4d, 4f and 4g inhibited the *in vitro* LPS-induced production of TNF-α in cultured mouse peritoneal macrophages at a concentration of 10 µM. Among them, 4f (93.2%, IC_50_ = 1.6 µM), 4a (96.9%, IC_50_ = 3.6 µM) and 4b (75.4%, IC_50_ = 4.3 µM) showed the most potent inhibitory effects. Compared with the unsubstituted phenyl ring compound 4g (cLogP = 5.3), the inhibitory potency increased when lipophilic groups [*para*-chloro for 4f (cLogP = 6.1), naphthyl for 4c (cLogP = 6.6), 4-hydroxynaphthyl for 4b (cLogP = 6.3) and 4-(2-(naphthalen-1-yloxy)ethyl)morpholine for 4a (cLogP = 6.0) were added. These results indicate that the differences in hydrophobicity of the imine-attached framework play an important role for the *in vitro* anti-TNF-α activity of *N*-phenylpyrazolyl-*N*-glycinyl-hydrazone derivatives.

**Table 2:** Effects of *N*-phenylpyrazolyl-*N*-glycinyl-hydrazone derivatives 4a-g on TNF-α production and cell viability in murine peritoneal macrophages.

Compound	TNF-α Inhibition^[a]^		% of Cell Viability [22]		cLogP[d]
	% inhibition at 10 µM	IC_50_ (µM)[b]	(at 3 µM)^[c]^	(at 10 µM)^[c]^	
4a	96.9*	3.6 (0.1–30)	100	61.5*	6.0
4b	75.4*	4.3 (0.1–30)	100	100	6.3
4c	61.2*	5.5 (0.1–30)	100	90.4	6.6
4d	52.5*	–	100	100	4.8
4e	8.1	–	100	97.8	5.1
4f	93.2*	1.6 (0.03–10)	100	46.6*	6.1
4g	50.4*	–	100	99.8	5.3
SB-203580 (1)	90.0*	0.22 (0.03–10)	100	44.3*	–

Results are expressed as percentage of inhibition**^[a]^** and percentage of cell viability**^[c]^** compared with vehicle (DMSO), n = 3 independent experiments in duplicate, *p<0.05 using student’s *t* test.

[b] IC_50_ were determined using at least five concentrations, the range concentration are showed in parentheses.

[d] Values calculated using ACDLABS program.

Because the novel *N*-acylhydrazone derivatives 4a–g were designed based on the p38α MAPK inhibitor BIRB-796 (3), they were all evaluated for their *in vitro* capacity to inhibit p38α MAPK activity [23] at a concentration of 10 µM. Interestingly, only compounds 4b and 4e were active, and they inhibited approximately 30% of p38α activity (Table S1).

**Figure 7:**
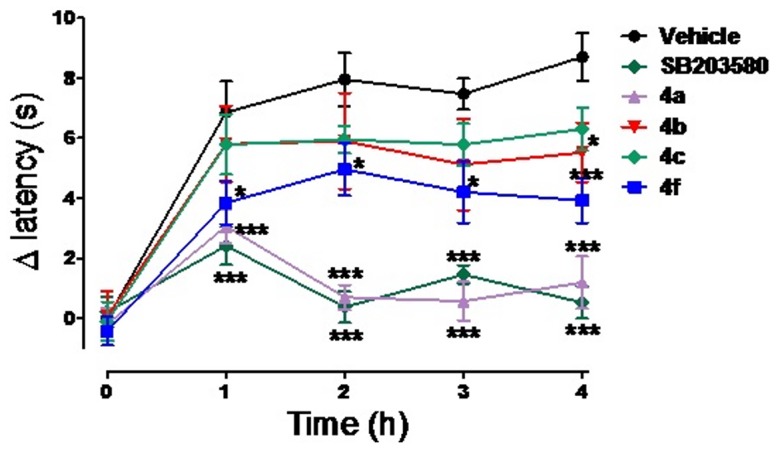
Effects of 4a, 4b, 4c, 4f and SB-203580 (100 µmol/kg, p.o.) on carrageenan-induced thermal hyperalgesia. n = 5–10 animals per group, the test groups were compared to the vehicle control group using two-way analysis of variance (ANOVA) followed by Bonferroni *post hoc* test, *p<0.05, ***p<0.001.

To evaluate the *in vivo* anti-inflammatory and antinociceptive profile of the NAH derivatives 4a, 4b, 4c and 4f, we employed the carrageenan-induced thermal hypernociception model [24]. Compounds were orally administered at a dose of 100 µmol/kg. SB-203580 (1) (100 µmol/kg, *p.o*.) was used as a standard. Figure 7 shows that compounds 4a and 4f were effective anti-hypernociceptive agents. Although these two compounds have shown similar capacities to inhibit TFN-α production *in vitro* (Table 2), compound 4a was more effective *in vivo*. In addition, compound 4a was able to completely inhibit the hypernociceptive response, whereas compound 4f was only able to partially inhibit this response.

**Figure 8:**
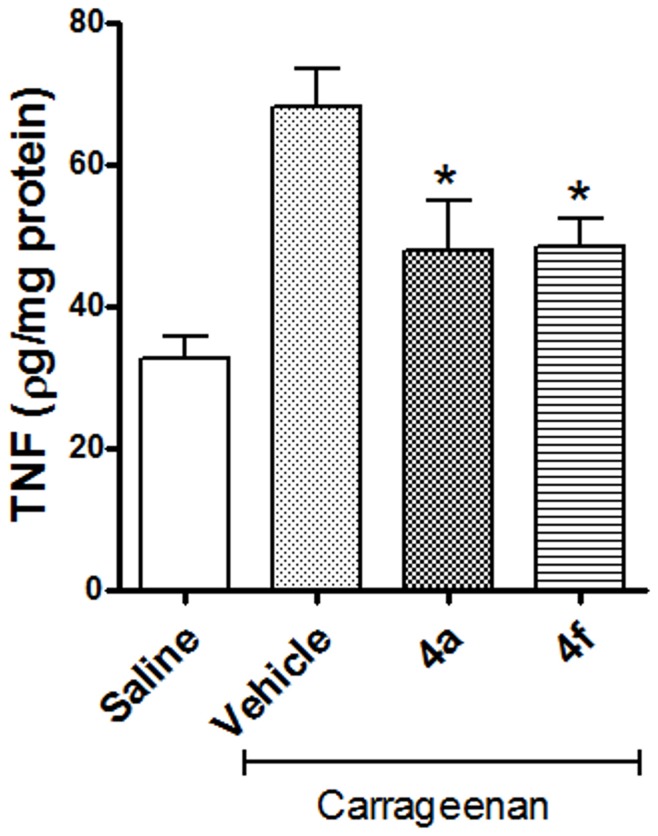
Effects of the NAH derivatives 4a and 4f (100 µmol/kg, p.o.) on the TNF-α level in carrageenan-injected paws. n = 8–10 animals per group, the test groups were compared to the vehicle control group using student’s *t* test, *p<0.05.

We then investigated whether the inhibition of carrageenan-induced thermal hypenociception by 4a and 4f occurs through the inhibition of TNF-α. Four hours after carrageenan injection, the TNF-α level in the paw was elevated by more than two times that of the saline control. Interestingly, pretreatment with 4a and 4f (100 µmol/kg) suppressed the elevation of tissue TNF-α level by 57.3 and 55.8%, respectively (Figure 8).

**Figure 9:**
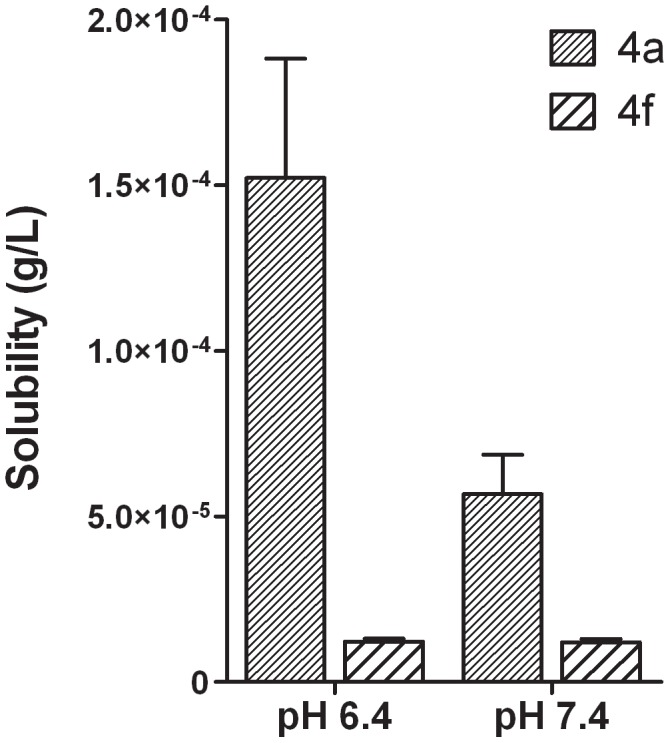
Aqueous solubility of compounds 4a and 4f in phosphate buffer at pH 6.4 and 7.4. Bars represent the mean ± S.E.M. of n = 3 independent measurements for each pH.

About the best anti-hypernociceptive profile of the compound 4a in comparison to derivative 4f, we decided to investigate the molecular reasons associated with a probable distinction in the respective pharmacokinetic behaviors. The physicochemical property cLog P doesn’t seems to explain the better *in vivo* profile of derivative 4a since both compounds, 4a and 4f, have the same theoretical lipophilicity, *i.e.* cLogP = 6.0 and 6.1, respectively. Considering that an adequate balance between the lipophilicity and aqueous solubility is essential for a good oral absorption of a drug candidate, we decided to determine experimentally the solubility of compounds 4a and 4f in buffer solutions of pH 6.4 and 7.4 (Figure 9). The derivative 4a, which contains the ethoxymorpholine-naphthyl group, exhibited an improvement in solubility at both pH values when compared with *para*-chlorophenyl derivative 4f, *i.e. ca.* 5 times at pH 7.4 and *ca.* 12 times at pH 6.4. As expected, at pH 6.4 only compound 4a showed to present an improvement in aqueous solubility (*ca.* three times), due to the partial ionization of its basic morpholine subunit. These solubility results allow us to rationalize that the improved *in vivo* activity of compound 4a is due to its better water solubility, which could favor its gastrointestinal absorption.

Moreover, we also evaluated the *in vitro* metabolic stability of derivatives 4a and 4f when placed in contact with preparations of liver and plasma of rats. The two NAH derivatives were resistant to oxidative microsomal metabolism, but the derivative 4a was about four times more resistant than derived 4f to plasma degradation, as described in Table 3. Taken together, these results indicate that the plasma stability associated to the better aqueous solubility are responsible for the better *in vivo* pharmacological profile shown by the NAH derivative 4a when given orally.

**Table 3:** *In vitro* stability of compounds 4a and 4f in rat liver microsome and rat plasma.

Compound(50 µM)	Rat liver microsomes	Rat plasma
	% Recovered amount after 60 min[a]	
4a	90.1	40.7
4f	86.7	13.3

[a] The percentage of compound remaining was calculated by ratio of peak area at 60 min to peak area found at 0 min multiplied by 100. The values are the mean of at least two experiments in duplicate.

This study describes the synthesis and pharmacological evaluation of novel *N*-phenylpyrazolyl-*N*-glycinyl-hydrazone derivatives that were designed as novel prototypes of p38 MAPK inhibitors. All novel synthesized compounds described were evaluated for their *in vitro* capacity to inhibit TNF-α production in cultured macrophages and their *in vitro* p38α MAPK inhibition. The two most active anti-TNF-α derivatives were (*E*)-2-(3-*tert*-butyl-1-phenyl-1H-pyrazol-5-ylamino)-*N*’-((4-(2-morpholinoethoxy)naphthalen-1-yl)methylene)acetohydrazide (4a) and (*E*)-2-(3-*tert*-butyl-1-phenyl-1H-pyrazol-5-ylamino)-*N*’-(4-chlorobenzylidene)acetohydrazide (4f). These two compounds were evaluated for their *in vivo* anti-hypenociceptive profiles. Both compounds showed anti-inflammatory and anti-hypenociceptive properties that were comparable to SB-203580 (1), which was used as a standard.

## Materials and Methods

Reactions were routinely monitored by thin-layer chromatography (TLC) in silica gel (F245 Merck plates) and the products visualized with ultraviolet lamp (254 and 365 nm). NMR spectra were recorded on a 200/50 MHz Bruker DPX-200, 250/62.5 MHz Bruker DPX-250, 400/100 MHz Varian 400-Mr, 300/75 MHz Varian Unity-300 spectrometer at room temperature. Peak positions are given in parts per million (δ) from tetramethylsilane as internal standard, and coupling constant values (*J*) are given in Hz. Infrared (IR) spectra were obtained using a Nicolet Magna IR 760 spectrometer. Samples were examined as potassium bromide (KBr) disks. Elemental analyses were carried out on a Thermo Scientific Flash EA 1112 Series CHN-Analyzer. Melting points were determined using a Quimis instrument and are uncorrected and the compounds 4a-f had their melting points determined using a differential scanning calorimeter (Shimadzu DSC-60). Column chromatography purifications were performed using silica gel Merck 230–400 mesh. All described products showed ^1^H and ^13^C NMR spectra according to the assigned structures.

All organic solutions were dried over anhydrous sodium sulfate and all organic solvents were removed under reduced pressure in rotatory evaporator.

HPLC for purity determinations were conducted using Shimadzu LC-20AD with a SHIM-PACK CLC-ODS analytical column (4.6 mm × 250 mm) or Kromasil 100-5C18 (4.6 mm × 250 mm) and a Shimadzu SPD-M20A detector at 254 nm wavelength. The solvent systems for HPLC purity analyses was acetonitrile:phosphate buffer solution pH7 = 70∶30. The isocratic HPLC mode was used, and the flow rate was 1.0 ml/min.

### Procedure for Preparation of 3-*tert*-butyl-1-phenyl-1H-pyrazol-5-amine (5)

A round-bottomed flask charged with phenylhydrazine (0.83 mL; 8.39 mmol), 4,4-dimethyl-3-oxo-pentanenitrile (2.0g; 8.0 mmol) and toluene (3 ml) was stirred and heated at reflux for 24 hours. The resulting mixture was concentrated on a rotary evaporator and the residue was purified by column chromatography on silica gel (hexane/ethyl acetate, gradient), to yield the title compound (1.38g, 80%) as a white solid (mp: 50–52°C). ^1^H NMR (200 MHz, DMSO-d_6_) δ = 7.59 (d, 2H, *J* = 8 Hz, H2 and H6-phenyl); 7.44 (t, 2H, *J* = 8 Hz, H3 and H5-phenyl); 7.26 (t, 1H, *J* = 8 Hz, H4-phenyl); 5.39 (s, 1H, CH-pyrazole); 5.19 (s, 2H, NH_2_); 1.22 (s, 9H, (CH_3_)_3_). ^13^C NMR (50 MHz, CDCl_3_, TMS) δ = 160.7 (C3-pyrazole), 146.9 (C5-pyrazole), 139.6 (C1-phenyl), 128.9 (C3 and C5-phenyl), 125.5 (C4-phenyl), 122.4 (C2 and C6-phenyl), 87.0 (CH-pyrazole), 31.8 (C(CH_3_)_3_), 30.2 (3xCH_3_). IR (KBr): 3412, 3284, 3146, 2961, 1597, 1556, 1507, 1382, 1243, 988, 696 cm^−1^.

### Procedure for the Preparation of Ethyl 2-(3-*tert*-butyl-1-phenyl-1H-pyrazol-5-ylamino)acetate (7)

To a solution of amine 5 (100 mg; 0.465 mmol) in toluene (3.0 mL) and trietylamine (0.1 mL), was added ethyl 2-bromoacetate (1.5 eq, 0.697 mmol, 0.077 mL). The resulting mixture was stirred and heated at reflux for 4 hours. The residue was partitioned between water and ethyl acetate. The combined organic phases were dried over Na_2_SO_4_, filtered, and concentrated. The brown residue was purified by silica gel chromatography hexane/ethyl acetate (gradient) to give the title compound (84 mg, 60%) as a brown oil. ^1^H NMR (200 MHz, CDCl_3_) δ = 7.57-7.44 (m, 4H, HAr); 7.31 (m, 1H, HAr); 5.71 (t, 1H, *J* = 6Htz, NH); 5.37 (s, 1H, CH-pyrazole); 4.12 (q, 2H, *J* = 8 Htz, CH_2_); 3.81 (d, 2H, CH_2_, *J* = 6 Hz); 1.21-1.19 (m, 12H, (CH_3_)_3_ and CH_3_).

### Procedure for the Preparation of 2-(3-*tert*-butyl-1-phenyl-1H-pyrazol-5-ylamino)acetohydrazide (8)

A round-bottomed flask charged with 600 mg (2 mmol) of ester (7), hydrazine hydrate 100% (20 eq) and ethanol (5 mL) was stirred and heated at reflux for 2 hours. To the resulting mixture was added water and the aqueous phase was extracted with ethyl acetate to give the title compound (430 mg, 80%) as a yellow oil. ^1^H NMR (200 MHz, DMSO-d_6_) δ = 9.17 (s, 1H, NHCO); 7.58 (d, 2H, *J* = 8,0 Hz, H2 and H6-phenyl); 7.47 (d, 2H, *J* = 8 Hz, H3 and H5-phenyl); 7.31 (t, 1H, *J* = 8 Hz, H4-phenyl); 5.56 (t, 1H, *J* = 6 Hz, NH); 5.35 (s, 1H, CH-pyrazole); 4.25 (s, 2H, NH_2_); 3.57 (d, 2H, *J* = 6 Hz, CH_2_); 1,37 (s, 9H, (CH_3_)_3_). ^13^C NMR (50 MHz, DMSO-d_6_) δ = 168.9 (CO), 160.9 (C3-pyrazole), 148.1 (C5-pyrazole), 139,2 (C1-phenyl), 129.1 (C3 and C5-phenyl), 126.1 (C4-phenyl), 123.2 (C2 and C6-phenyl), 84,4 (CH-pyrazole), 47.5 (CH_2_), 31.2 (C(CH_3_)_3_), 30.2 (CH_3_). IR (NaCl) 3319, 2960, 1669, 1596, 1567, 1520, 1373, 1247, 991, 764 cm^−1^.

### General Procedure for the Preparation of *N*-phenyl-pyrazole *N*-glycinyl-*N*-acylhydrazones (4a–g)

In a round flask containing hydrazide 8 (1.6 mmol) in ethanol (10 mL), was added aldehyde (1.68 mmol; 1.05 eq) and catalytic concentrated hydrochloric acid. The mixture was stirred for about 2 hours at room temperature. At the end of the reaction the volume of ethanol was reduced, saturated solution of sodium bicarbonate and ice were added to the reaction. The precipitate formed was filtered, or the mixture was extracted with dichloromethane.

### (*E*)-2-(3-tert-butyl-1-phenyl-1H-pyrazol-5-ylamino)-*N*’-((4-(2-morpholinoethoxy)naphthalen-1-yl)methylene)acetohydrazide (4a)

(Yield = 75%) (mp. 180°C).

^1^H NMR (300 MHz, DMSO-d_6_) δ = 11.37 and 11.28 (s, 1H, NHCO); 8.91 and 8.72 (d, 1H, *J* = 9 Hz, CH-naphthyl); 8.72 and 8.53 (s, 1H, N = CH); 8.26 (m, 1H, CH-naphthyl); 7.77 (m, 1H, CH-naphthyl); 7.40-7.60 (m, 6H, ArH); 7.33 (m, ArH); 7.04 (d, 1H, *J* = 9 Hz, ArH); 5.64 and 5.35 (t, 1H, *J* = 5.7 Hz, NH); 5,45 (s, CH-pyrazole); 4.33 (t, 2H, *J* = 4,6 Hz, OCH_2_-ethoxyl); 4.24 and 3.78 (d, 2H, *J* = 5,7 Hz, CH_2_); 3.56 (t, 4H, *J* = 4,7 Hz, 2xCH_2_-morpholine); 2.88 (t, 2H, *J* = 4,7 Hz, CH_2_-ethoxyl); 2.56 (t, 4H, *J* = 4,7 Hz, 2xCH_2_-morpholine); 1.23 (s, 9H, (CH_3_)_3_) [Figure S1]. ^13^C NMR (50MHz, DMSO, TMS) δ = 170.6 and 166.1 (C = O); 161.0 (C3-pyrazol); 155.7 (C4-naphthyl); 148.2 (C5-pyrazol); 147.7 and 144.3 (N = CH); 139.2 (C1-phenyl); 131.0 (C9-naphthyl); 129.2 (ArCH); 127.8 (ArCH); 126.2 (ArCH); 125.7 (ArCH); 125.2 (ArCH); 124.6 (Ar CH); 124.0 (ArCH); 123.1 (C1-nafhtyl); 122.3 (ArCH); 121.7 (C10-naphthyl); 105.2 (ArCH); 84.5 (CH-pyrazole); 66.4 (OCH_2_-ethoxyl); 66.2 (CH_2_-morfolyn); 56.9 (CH_2_-ethoxyl); 53.6 (CH_2_-morpholine); 48.0 and 46.5 (CH_2_); 31.9 (C(CH_3_)_3_); 30.2 (3xCH_3_) [Figure S2]. IR (KBr): 3468, 3175, 2964, 1654, 1572, 1397, 1228, 1120, 764 cm^−1^. % purity >98% by HPLC. Anal. Calcd for C_32_H_38_N_6_O_3_H_2_O: C 67.11; H 7.04; N 14.67. Found: C 67.16; H 7.05; N 14.74.

### (*E*)-2-(3-*tert*-butyl-1-phenyl-1H-pyrazol-5-ylamino)-*N*’-((4-hydroxynaphthalen-1-yl)methylene)acetohydrazide (4b)

(Yield = 70%) (mp. 270°C).

^1^H NMR (200 MHz, DMSO-d_6_) δ = 11.39 and 11.28 (s, 1H, CO); 10.78 (s, 1H, OH); 8.93 and 8.72 (d, 1H, *J* = 8 Hz, H-Ar); 8.73 and 8.52 (s, 1H, N = CH); 8.25 (d, 1H, *J* = 8 Hz, H-Ar); 7.74-7.36 (m 8H, HAr); 5.74 and 5.42 (t, 1H, *J* = 6 Hz, NH); 5.49 and 5.46 (s, 1H, CH-pyrazole); 4.25 and 3.80 (d, 2H, *J* = 6 Hz, CH2); 1.23 (s, 9H, (CH_3_)_3_) [Figure S3]. ^13^C NMR (50 MHz, CDCl_3_) δ = 171.1 and 166.0 (CO); 161.6 (C3-pyrazole); 156.1 (C-OH); 148.8 (N = CH); 145.2 (C5-pyrazole); 139.8 (C1-phenyl); 132.04 (C9-naphthyl); 130.4 (C2-naphthyl); 129.8 (C3 and C5-phenyl); 128.2 (C5-naphthyl); 126.7 (C4 phenyl); 125.6 (C1-naphthyl); 125.3 (C7-naphthyl); 124.4 (C2 and C6-phenyl); 123.8 (C10-naphthyl); 123.3 (C6-naphthyl); 120.7 (C8-naphthyl); 108.6 (C3-naphthyl); 85.0 (CH-pyrazole); 47.0 (CH_2_); 32.5 (C(CH_3_)_3_); 30.8 (CH_3_)_3_) [Figure S4]. IR (KBr): 3396, 3186, 2956, 2903, 2865, 1678, 1571, 1523, 1287, 1169, 759, 698 cm^−1^. % purity >98% by HPLC. Anal. Calcd for C_26_H_27_N_5_O_2_: C 70.73; H 6.16; N 15.86. Found: C 70.25; H 6.20; N 15.52.

### (*E*)-2-(3-*tert*-butyl-1-phenyl-1H-pyrazol-5-ylamino)-*N*’-(naphthalen-1-ylmethylene)acetohydrazide (4c)

(Yield = 90%) (mp. 140°C).

^1^H NMR (200 MHz, DMSO-d_6_) δ = 11.60 and 11.53 (s, 1H, NHCO); 8.90 and 8.69 (s, 1H, N = CH); 8.84 and 8.64 (d, 1H, *J* = 8,0 Hz, H8-naphthyl); 8.03-7.88 (m, 3H, HAr); 7.63-7.49 (m, 7H, HAr); 5.78 and 5.45 (t, 1H, *J* = 6,0 Hz, NH); 5.50 (s, 1H, CH-pyrazole); 4.30 e 3.84 (d, 2H, *J* = 6,0 Hz, CH2); 1.23 (s, 9H, (CH3)3) [Figure S5]. ^13^C NMR (50 MHz, DMSO-d_6_) δ = 171.5 and 167.1 (CO); 161.6 (C3-pyrazole); 148.7 (C5-pyrazol); 147.6 and 144.1 (HC = N); 139.8 (C1-phenyl); 134.1 (C1-naphthyl); 131.0 (C10-naphthyl); 130.6 (C4-naphthyl); 129.8 (C3 e C5-phenyl); 129.4 (C5-naphthyl); 128.5 (C4-phenyl); 127.9 (C2-naphthyl); 126.8 (C7-naphthyl); 126.1 (C2 and C6-phenyl); 124.8 (C6-naphthyl); 124.2 (C9-naphthyl); 123.7 (C8-naphthyl); 85.1 (CH-pyrazole); 46.6 and 47.0 (CH_2_); 32.49 (C(CH_3_)_3_); 30.75 ((CH_3_)_3_) [Figure S6]. IR (KBr): 3389, 3196, 2948, 1680, 1588, 1284, 1162, 974, 751, 702 cm^−1^. Anal. Calcd for C_26_H_27_N_5_O: C 73.39; H 6.40; N 16.46. Found: C 73.07; H 6.41; N 16.17.

### (*E*)-2-(3-*tert*-butyl-1-phenyl-1H-pyrazol-5-ylamino)-*N*’-(4-(2-morpholinoethoxy)benzylidene)acetohydrazide (4d)

(Yield = 60%) (mp. 158°C).

^1^H NMR (200 MHz, DMSO-d_6_) δ = 11.71 and 11.56 (s, 1H, NHCO); 8.10 and 7.90 (s, 1H, N = CH); 7.65-7.49 (m, 6H, H-Ar); 7.30 (t, 1H, H-Ar); 7.00 (d, 2H); 5.47 and 5.40 (s, 1H, CH-pyrazole); 4.14-3.60 (m, 4H, H-Ar); 3.57 (m, 4H, H-Ar); 2.69 (t, 2H, H-Ar); 2.49 (m, 2H, H-Ar); 1.23 (s, 9H, (CH_3_)_3_) [Figure S7]. ^13^C NMR (50 MHz, DMSO-d_6_) δ = 171.3 and 166.7 (C = O); 161.6 (C3-pyrazole); 160.6 (C4-phenyl); 148.7 and 147.5 (N = CH); 144.3 (C5-pyrazole); 139.8 (C1-phenylpyrazol); 129.8 (C3 and C5-phenylpyrazole); 129.0 (C2 and C6-phenylpirazole); 127.3 (C1-phenyl); 126.7 (C4-phenylpyrazole); 123.6 (C2 and C6-phenyl); 115.4 (C3 and C5-phenyl); 85.0 (CH-pyrazole); 66.7 (CH_2_-morfolyn); 66.0 (OCH_2_-ethoxyl); 57.5 (CH_2_-ethoxyl); 54.2 (CH_2_-morfolyn); 46.8 (CH_2_); 32.5 (C(CH_3_)_3_); 30.8 (3xCH_3_) [Figure S8]. IR (KBr): 3440, 2959, 1673, 1595, 1399, 1285, 1120, 758 cm^−1^. Anal. Calcd for C_28_H_36_N_6_O_3_: C 66.64; H 7.19; N 16.65. Found: C 66.10; H 7.13; N 16.51.

### (*E*)-2-(3-*tert*-butyl-1-phenyl-1H-pyrazol-5-ylamino)-*N*’-(4-hydroxybenzylidene)acetohydrazide (4e)

(Yield = 70%) (mp. 280°C).

^1^H NMR (200 MHz, DMSO-d_6_) δ = 11.36 and 11.23 (s, 1H, NHCO); 9.93 (s, 1H, OH); 8.14 and 7.91 (s, 1H, N = CH); 7.64-7.46 (m, 6H, HAr); 7.36 (m, 1H, HAr); 6.83 (d, 2H, *J* = 8.5 Hz, H-Ar); 5.70 and 5.37 (t, 1H, *J* = 5.7 Hz, NH); 5.47 and 5.41 (s, 1H, CH-pyrazole); 4.16 and 3.74 (d, 2H, CH_2_, *J* = 5.7 Hz); 1.23 (s, 9H, (CH_3_)_3_) [Figure S9]. ^13^C NMR (50 MHz, DMSO-d_6_) δ = 171.4 and 166.6 (CO); 161.6 (C3-pyrazole); 159.9 (C4-OH); 148.7 and 144.7 (HC = N); 147.9 (C5-pyrazole); 139.8 (C1-phenyl); 129.8 (C2 and C6-phenol); 129.3 (C3 and C5-phenyl); 126.7 (C4-phenyl); 125.7 (C1-phenyl); 123.7 (C2 and C6-phenyl); 116.2 (C3 and C5-phenol); 85.1 and 85.0 (CH-pyrazole); 48.5 and 46.8 (CH_2_); 31.2 (C(CH_3_)_3_); 30.8 ((CH_3_)_3_) [Figure S10]. IR (KBr): 3348, 3066, 2959, 1684, 1604, 1574, 1524, 1285, 1163, 835, 695 cm^−1^. Anal. Calcd for C_22_H_24_N_5_O_2_: C 67,50; H 6,44; N 17,89. Found: C 67,27; H 6,33; N 17,88.

### (*E*)-2-(3-*tert*-butyl-1-phenyl-1H-pyrazol-5-ylamino)-*N*’-(4-chlorobenzylidene)acetohydrazide (4f)

(White solid; recr. from ethanol; Yield = 70%) (mp. 193°C).

^1^H NMR (200 MHz, DMSO-d_6_) δ = 11.62 and 11.60 (s, 1H, NHCO); 8.27 and 8.01 (s, 1H, N = CH); 7.75-7.30 (m, 9H, H-Ar); 5.53 and 5.46 (s, 1H, CH-pyrazole); 4.21 and 3.78 (s, 2H, CH_2_); 1.23 (s, 9H, (CH_3_)_3_) [Figure S11]. ^13^C NMR (50 MHz, CDCl_3_) δ = 171.6 (CO); 161.50 (C3-pyrazole); 148.9 (C5-pyrazole); 143.1 (HC = N); 139.1 (C1-phenylpyrazole); 134.9 (C4-phenyl); 133.5 (C1-phenyl); 129.8 (2xCH-Ar); 129.4 (2xCH-Ar); 129.2 (2xCH-Ar); 127.0 (CH-Ar); 123.9 (2xCH-Ar); 85.3 (CH-pyrazole); 46.7 (CH_2_); 32.5 (C(CH_3_)_3_); 30.7 ((CH_3_)_3_). IR (KBr): 3374, 2950, 1687, 1593, 1511, 1397, 1284, 984, 757 cm^−1^. Anal. Calcd for C_22_H_24_ClN_5_O: C 64.46; H 5.90; N 17.09. Found: C 64.04; H 5.81; N 16.68.

### (*E*)-*N*’-benzylidene-2-(3-*tert*-butyl-1-phenyl-1H-pyrazol-5-ylamino)acetohydrazide (4g)

(White solid; Yield = 70%; mp. 148°C).

^1^H NMR (300 MHz, DMSO-d_6_) δ = 11.49 and 11.38 (s, 1H, NHCO); 8.27 and 8.02 (s, 1H, N = CH); 7.60-7.30 (m, 10H, H-Ar); 5.49 and 5.43 (s, 1H, CH-pyrazole); 4.20 and 3.77 (d, 2H, *J* = 5.7 Hz, CH_2_); 1.23 (s, 9H, (CH_3_)_3_) [Figure S12]. ^13^C NMR (50 MHz, CDCl_3_) δ = 172.9 and 166.3 (CO); 160.9 (C3-pyrazole); 148.0 (C5-pyrazole); 147.0 and 143.8 (CH = N); 139.2 (C1-phenylpyrazole); 133.9 (C1-phenyl); 129.8 (CH-Ar); 129.2 (2xCH-Ar); 128.7 (2xCH-Ar); 126.8 (2xCH-Ar); 126.0 (CH-Ar); 123.0 (2xCH-Ar); 84.4 (CH-pyrazole); 47.9 and 46.2 (CH_2_); 31.9 (C(CH_3_)_3_); 30.1 ((CH_3_)_3_) [Figure S16]. IR (KBr): 3446, 2952, 1684, 1595, 1278, 753 cm^−1^. % purity >98% by HPLC. Anal. Calcd for C_22_H_25_N_5_O: C 70.38; H 6.71; N 18.65. Found: C 70.26; H 6.65; N 18.42.

### Procedure for the Preparation of (*E*)-*N*’-benzylidene-2-(3-*tert*-butyl-1-phenyl-1H-pyrazol-5-ylamino)-*N*-methylacetohydrazide (4h)

Iodomethane (1.5mmol) was added dropwise to the magnetically stirred solution of 8g (375 mg, 1 mmol) in acetone 50 mL and K_2_CO_3_ (1.5 mmol). The mixture was stirred and maintained at 40°C. The reaction was monitored by TLC. After the reaction was complete, the acetone was evaporated on a rotatory evaporator, ethanol was added and then ice. The solid was filtered and washed with water to give compound 4h (233 mg, 60%). ^1^H NMR (400 MHz, DMSO-d_6_) δ = 8.01 (s, 1H, N = CH); 7.78 (d, 2H, *J* = 8 Hz, ArH); 7.59 (d, 2H, *J* = 8 Hz, HAr); 7.51-7.44 (m, 5H, HAr); 7.32 (t, 1H, *J* = 8 Hz, HAr); 5.47 (s, CH-pyrazole); 5.39 (t, 1H, *J* = 6 Hz, NH); 4.34 (d, 2H, *J* = 6 Hz, CH_2_); 3.34 (s, 3H, CH_3_); 1.22 (s, 9H, (CH_3_)_3_).

### Procedure for the Preparation of 2-(3-*tert*-butyl-1-phenyl-1H-pyrazol-5-ylamino)-*N*’-(propan-2-ylidene)acetohydrazide (9)

A solution of 1 mmol of 2-(3-*tert*-butyl-1-phenyl-1H-pyrazol-5-ylamino)acetohydrazide (8) in 60 mL of acetone was refluxed for 3 hours. Then half of the volume was evaporated on a rotatory evaporator and the residue was cooled in the refrigerator for 3 hours. The crystals which formed were filtered, dried and recrystallized from acetone (262 mg, 80%, mp: 181–183°C). ^1^H NMR (200 MHz, DMSO-d_6_) δ = 10.33 and 10.04 (s, 1H, NHCO); 7.60-7.46 (m, 4H, ArH); 7.32 (m, 1H, HAr); 5.38 and 5.41 (s, CH-pyrazole); 5.59 and 5.29 (t, 1H, *J* = 6 Hz, NH); 4,03 and 3.74 (d, 2H, *J* = 6 Hz, CH_2_); 1.93, 1.86 and 1.83 (s, 6H, 2xCH_3_); 1.23 (s, 9H, (CH_3_)_3_) [Figure S17].

### Procedure for Preparation of *N*-(3-*tert*-butyl-1-phenyl-1H-pyrazol-5-yl)acetamide (10)

A solution of 0.3g (3.66 mmol) of sodium acetate anhydrous in 1.0 mL (17.5 mmol) of glacial acetic acid was prepared. 0.85g of amine 5 (3.95 mmol) was slowly added, and then 1.0 ml (10.6 mmol) of acetic anhydride was also added. The reaction mixture was stirred for about 3 h, at room temperature, when the end of reaction was observed by TLC. Water was added and the mixture was extracted with ethyl acetate. The *N*-(3-*tert*-butyl-1-phenyl-1H-pyrazol-5-yl)acetamide (10) was obtained as a powder (812 mg, 80%, mp: 88–90 °C) after concentration on a rotary evaporator. ^1^H NMR (200 MHz, DMSO-d6) δ = 9.83 (s, 1H, NHCOCH3); 7.46-7.38 (m, 5H-phenyl); 6.29 (s, 1H, CH-pyrazole); 1.96 (s, 3H, NHCOCH_3_); 1.28 (s, 9H, (CH_3_)_3_). ^13^C NMR (50 MHz, DMSO-d_6_) δ = 168.8 (CO); 160.7 (C3-pyrazole); 138.9 (C1-phenyl); 136.0 (C5-pyrazole); 129.0 (C3 and C5-phenyl); 126.9 (C4-phenyl); 123.3 (C2 and C6-phenyl); 99.0 (C4-pyrazole); 32.0 (C(CH_3_)_3_); 30.1((CH_3_)_3_); 22.8 (COCH_3_). IR (KBr): 3255, 3218, 3058, 2946, 1671, 1554, 1501, 1373, 1399, 1277, 1235, 767, 687 cm^−1^.

### Procedure for Preparation of *N*-(3-*tert*-butyl-1-phenyl-1H-pyrazol-5-yl)-*N*-methylacetamide (11)

Sodium hydride (100 mg, 4.2 mmol) was added to the magnetically stirred acetamide 10 (900 mg, 3.5 mmol) in anhydrous THF (5 mL), and iodomethane (0,26 mL, 4.2 mmol) was added dropwise to the mixture, which was maintained below 5°C for 0.5 h, and stirred at room temperature. The reaction was monitored by TLC. After the reaction was complete, the reaction mixture was partitioned between saturated aqueous NH_4_Cl and ethyl acetate. The organic layer was separated and the aqueous layer was extracted with ethyl acetate. The combined organic layers were washed with water, dried over Na_2_SO_4_, and concentrated to give the *N*-(3-*tert*-butyl-1-phenyl-1H-pyrazol-5-yl)-*N*-methylacetamide (11) as a solid in (854 mg, 90%): mp: 108–110°C. ^1^H NMR (200 MHz, DMSO-d_6_) δ = 7.56-7.39 (m, 5H-phenyl); 6.48 (s, 1H, CH-pyrazole); 3.01 (NCH_3_COCH_3_); 1.67 (NCH_3_COCH_3_); 1.30 (s, 9H, (CH_3_)_3_). ^13^C NMR (50 MHz, DMSO-d_6_) δ = 169.7 (CO); 161.5 (C3-pyrazol); 141.6 (C5-pyrazole); 138.3 (C1-phenyl); 129.4 (C3 and C5-phenyl); 127.5 (C4-phenyl); 122.8 (C2 and C6-phenyl); 101.0 (C4-pyrazole); 35.8 (NCH_3_COCH_3_); 35.8((C(CH_3_)_3_); 30.0 ((CH_3_)_3_); 21.4 (NCH_3_COCH_3_). IR (KBr): 2969, 2867, 1677, 1594, 1566, 1502, 1366, 1334, 758, 688, 655 cm^−1^.

### Procedure for Preparation of 3-*tert*-butyl-*N*-methyl-1-phenyl-1H-pyrazol-5-amine (12)

Concentrated HCl (0.25 ml) was added to a stirred solution of *N*-(3-*tert*-butyl-1-phenyl-1H-pyrazol-5-yl)-*N*-methylacetamide (11) (1 mmol) in ethylene glycol (0.75 ml). The reaction mixture was heated to reflux and the reaction was monitored by TLC. When the reaction was completed, saturated sodium bicarbonate solution was added and the reaction mixture was partitioned between water and ethyl acetate. The combined organic layers were washed with water, dried over Na_2_SO_4_, and concentrated to give 3-*tert*-butyl-*N*-methyl-1-phenyl-1H-pyrazol-5-amine (12) as oil (290 mg, 90%). ^1^H NMR (200 MHz, DMSO-d6) δ = 7.55-7.23 (m, 5H-phenyl); 5.42 (s, 1H, CH-pyrazole); 5.33 (NH); 2.65 (d, 3H, J = 4 Hz, NCH3); 1.24 (s, 9H, (CH_3_)_3_). ^13^C NMR (50 MHz, DMSO-d6) δ = 161.1 (C3-pyrazole); 150.4 (C5-pyrazole); 139.6 (C1-phenyl); 129.1 (C3 and C5-phenyl); 125.8 (C4-phenyl); 122.8 (C2 and C6-phenyl); 84.2 (C4-pyrazole); 32.1 (NCH_3_); 32.0((C(CH_3_)_3_); 30.2 ((CH_3_)_3_). IR (NaCl) 3370, 2958, 1594, 1570, 1520, 1498, 1375, 1244, 760, 697 cm^−1^.

### Procedure for preparation of ethyl 2-((3-*tert*-butyl-1-phenyl-1H-pyrazol-5-yl)(methyl)amino)acetate (13)

Ethyl 2-bromoacetate (0.75 ml, 6.8 mmol, 2.0 equiv) was added slowly at room temperature to a solution of 3-*tert*-butyl-*N*-methyl-1-phenyl-1H-pyrazol-5-amine (12) (3.4 mmol, 1.0 equiv) in EtOH (30 ml). The reaction mixture was stirred at room temperature for 30 min before Na_2_CO_3_ (1.08g, 10.2 mmol, 3.0 equiv) was added and refluxed overnight. After cooling to room temperature, the solution was concentrated by evaporation and the residue was partitioned between water and ethyl acetate. The combined organic phases were washed with water and saturated NaCl solution, dried over Na_2_SO_4_, filtered, and concentrated. The brown residue was purified by silica gel chromatography hexane/ethyl acetate (gradient) to give the title compound (642 mg, 60%) as a brown oil. ^1^H NMR (200 MHz, DMSO-d_6_) δ = 7.69 (d, 2H, *J* = 8 Hz, H2 and H6-phenyl); 7.44 (t, 2H, *J* = 8 Hz, H3 and H5-phenyl); 5.86 (s, 1H, CH-pyrazole); 3.68 (s, CH2); 3.48 (q, 2H, *J* = 6 Hz, CH_2_); 2.64 (s, 3H, NCH_3_); 1.34 (s, 9H, (CH_3_)_3_); 0.95 (m, 3H, CH_3_). ^13^C NMR (50 MHz, DMSO-d_6_) δ = 169.4 (CO); 160.5 (C3-pyrazol); 150.8 (C5-pyrazol); 140.0 (C1-phenyl); 128.9 (C3 and C5-phenyl); 126.2 (C4-phenyl); 122.6 (C2 and C6-phenyl); 91.6 (C4-pyrazole); 65.9 (CH_2_ ester); 60.0 (CH_2_ spacer); 41.1 (NCH_3_); 31.9((C(CH_3_)_3_); 30.0 ((CH_3_)_3_); 14.0 (CH_3_ ester). IR (NaCl): 2964, 2870, 1749, 1595, 1556, 1502, 1454, 1373, 1198, 1137, 1030, 765, 693 cm^−1^.

### Procedure for Preparation of 2-((3-*tert*-butyl-1-phenyl-1H-pyrazol-5-yl)(methyl)amino)acetohydrazide (14)

A round-bottomed flask charged with 472.5 mg (1.5 mmol) of ester (13), hydrazine hydrate 100% (20 eq) and ethanol (5 mL) was stirred and heated at reflux for 2 hours. To the resulting mixture was added water and the aqueous phase was extracted with dichloromethane to give the title compound (361 mg, 80%) as a yellow oil after solvent concentration. ^1^H NMR (200 MHz, DMSO-d_6_) δ = 9.00 (s, 1H, NHCO); 7.71 (m, 2H, H2 and H6-phenyl); 7.38 (m, 2H, H3 and H5-phenyl); 7.24 (m, 1H, H4-phenyl); 5.82 (s, 1H, CH-pyrazole); 4.18 (s, 2H, CH_2_); 2.56 (NCH_3_); 1.19 (s, 9H, (CH_3_)_3_). ^13^C NMR (50 MHz, DMSO-d_6_) δ = 167.7 (CO), 160.5 (C3-pyrazole), 151.5 (C5-pyrazol), 140.0 (C1-phenyl), 128.9 (C3 and C5-phenyl), 126.1 (C4-phenyl), 122.7 (C2 and C6-phenyl), 91.7 (CH-pyrazole), 56.4 (CH_2_), 44.0 (NCH_3_), 32.0 (C(CH_3_)_3_), 30.2 (CH_3_). IR (NaCl): 3315, 2960, 2862, 1673, 1598, 1502, 1553, 1371, 1271, 1116, 1018, 767 cm-1.

### Procedure for Preparation of (*E*)-*N*’-benzylidene-2-((3-*tert*-butyl-1-phenyl-1H-pyrazol-5-yl)(methyl)amino)acetohydrazide (15)

In a round flask containing hydrazide 14 (0.93 mmol) in ethanol (5 ml), was added benzaldehyde (0.1 ml; 0.97 mmol; 1.05 eq) and catalytic concentrated hydrochloric acid. The mixture was stirred for about 2 hours at room temperature. At the end of the reaction the volume of ethanol was reduced, saturated solution of sodium bicarbonate and ice were added to the reaction and the mixture was extracted with dichloromethane. The combined organic phases were dried over Na_2_SO_4_, filtered, and concentrated. The white solid obtained was washed with n-hexane and filtered under vacuum to give the title compound as a white solid (217 mg, 60%): mp: 156–158°C. ^1^H NMR (200 MHz, DMSO-d_6_) δ = 11.34 (s, 1H, NHCO); 8.35 and 8.18 (s, 1H, N = CH); 7.89-7.20 (m, 10H, H-Ar); 5.93 and 5.87 (s, 1H, CH-pyrazole); 4.06 and 3.61 (s, 2H, CH2); 2.72 and 2.65 (s, 3H, CH3);1.23 (s, 9H, (CH3)3). ^13^C NMR (50 MHz, CDCl_3_) δ = 170.1 and 165.0 (CO); 160.5 (C3-pyrazole); 151.4 (C5-pyrazole); 151.7 and 146.8 (N = CH); 143.1 (C1-phenylpyrazole); 140.2 and 140.0 (C1-phenyl); 134.1 and 134.0 (C4-phenyl); 130.1 (C4-phenylpyrazole); 128.8 and 128.7 (C3 and C5-phenylpyrazole); 127.0 and 126.7 (C3 and C5-phenyl); 126.2 and 126.0 (C2 and C6-phenylpyrazole); 122.9 and 122.6 (C2 and C6-phenyl); 91.9 and 91.3 (CH-pyrazole); 56.6 and 54.8 (CH_2_); 41.5; 32.0 (C(CH_3_)_3_); 30.1 ((CH_3_)_3_). IR (KBr): 3196, 3080, 2957, 2857, 1682, 1592, 1500, 1402, 1314, 1267, 759, 693 cm^−1^.

### Animals

Animals were obtained from the LASSBio breeding unit (Faculty of Pharmacy, UFRJ, Brazil). All animals were kept under standardized conditions, maintained in a 12-h light/dark cycle with water and food ad libitum until use. Animal experiments were performed according to the “Principles of Laboratory Animal Care and Use in Research” (Colégio Brasileiro de Experimentação Animal-COBEA/Instituto Brasileiro Carlos Chagas Filho-IBCCF, Brazil), based on international guidelines for the care and use of laboratory animals. All experiments were previously approved by the local ethics committee.

### LPS-induced TNF-α Production in Culture of Mice Peritoneal Macrophage

BALBc mice were stimulated with thyoglicollate 3% (1 mL/mice; i.p.) and 3 days later the peritoneal cavity were washed with RPMI 1640 and the peritoneal macrophages were plated onto 96-wells plate (30.000 cells/well) for 1 hour at 37°C in an humidified 5% CO_2_ atmophere. Then, macrophages were incubated with the vehicle or compounds and 1 hour later stimulated with LPS (100 ng/mL) for 24 hour when the supernatants were collected to evaluate TNF-α production by ELISA kit (BD Bioscience).

### Cell viability by MTT Assay

The peritoneal macrophage were obtained and plated as described above. The cells were incubated with the vehicle or compounds for 20 hours when was added 20 µl of MTT (3-(4,5-dimethylthiazol-2-yl)-2,5-diphenyltetrazolium bromide) solution (5 mg/mL), followed by 4 hour of incubation. Then, the culture mediums were collected and the precipitates were solubilized in 200 µl of DMSO. The optical density was measured at 490 nm.

### Carragenaan-induced Hypernociception Assay

Wistar rats of both sexes (150–200g) were used. The compounds were administered orally (100 µmol.5mL-1.kg-1) as a suspension in 5% arabic gum in saline (vehicle). Control animals received an equal volume of vehicle. One hour later, the animals were injected with either 0.1 ml of 1% carrageenan solution in saline or sterile saline (NaCl 0.9%), into the subplantar surface of one of the hind paws. The thermal hypernociception was determined using the modified hot-plate test. Rats are placed individually on a hot plate with the temperature adjusted to 51°C. The latency of the withdrawal response of the left hind paw is determined at 0, 30, 60, 120, 180, and 240 min post-challenge. The time of maximum permanence permitted on the hot surface is 20 s. Hypernociception to heat is defined as a decrease in withdrawal latency and calculated as follows: Δ paw withdrawal latency (s) = (left paw withdrawal latency at time 0) – (left paw withdrawal latency at the others times).

The paw was homogenized 4 h after intraplantar injection of carrageenan, and the level of TNF-α in the supernatant was determined by ELISA.

### Solubility

The solubility was evaluated after twenty-four hour agitation of 1 mg of test compound in 1 mL aqueous buffer (pH 6.4 and pH 7.4) at 37 °C followed by centrifugation and filtration for HPLC-UV analysis.

### Rat liver Microsomal Stability Studies

The incubation was conducted at 37°C for 60 min. The experiments contains MgCl_2_ (1.3 mM), NADP+ (0.4 mM), glucose-6-phosphate (3.5 mM), 0.5 U/mL glucose-6-phosphate dehydrogenase in a phosphate buffer (0.1 M, pH 7.4) containing EDTA (1.5 mM) and the test compounds were added at final concentration of 50 µM with 0.25 mL of final volume. After the pre-warming of the mixture at 37 °C, the microsomal proteins were added to give a final protein concentration of 1 mg/mL. At the end of the incubation time the reaction was stopped by the addition of 375 µL of MeOH and 375 µL of CH_3_CN. The experiments were performed in duplicate. The samples were centrifuged and filtered for HPLC-UV analysis.

### Rat Plasma Stability Studies

The rat plasma was obtained from blood by centrifugation and diluted in phosphate buffer (pH 7.4). The test compounds were added at final concentration of 50 µM with 0.25 mL of final volume and incubated at 37°C for 60 min under agitation. At the end of the incubation time the reaction was stopped by the addition of 375 µL of MeOH and 375 µL of CH_3_CN. The experiments were performed in duplicate. The samples were centrifuged and filtered for HPLC-UV analysis.

### HPLC-UV Analysis

The organic fraction was analyzed with the Shimadzu Prominence HPLC system (Shimadzu, Tokio, Japan) consisting of a vacuum degasser (DGU-20A5), a binary pump (LC-20AD), a autosampler (SIL-20A), UV/VIS Photodiode Array Detector (SPD-M20A) and fitted with a guard column (CLC G-ODS) and a Shimadzu (CLC-ODS, M) column (250 mm×4.6 mm i.d.) running at room temperature. Isocratic elution was performed with acetonitrile-water (40∶60 v/v), at a flow rate set at 1 mL/min. The mobile phase pH was adjusted to 8.0 with NH_4_OH solution. The detection was carried out at 285 nm and 330 nm wavelength for compound 4f and 4a, respectively.

### Statistical Analysis

Data obtained from experiments were expressed as mean ± S.E.M., compared with vehicle control groups and statistically analyzed by the ANOVA one-way (Bonferroni *post hoc* test) for carrageenan-induced thermal hyperalgesia and Student’s *t* test or for the others experiments. In all cases p<0.05 was considered significant (*p<0.05; **p<0.01; ***p<0.001). When appropriate, the IC_50_ values (i.e. the concentration able to inhibit 50% of the maximum effect observed) were determined by non-linear regression using GraphPad Prism software v. 5.0.

## Supporting Information

Figure S1 ^1^H NMR spectrum of 4a (DMSO-d_6_, 300 MHz).
(TIF)

Figure S2 ^13^C NMR spectrum of 4a (DMSO-d_6_, 75 MHz).
(TIF)

Figure S3 ^1^H NMR spectrum of 4b (DMSO-d_6_, 200 MHz).
(TIF)

Figure S4 ^13^C NMR spectrum of 4b (DMSO-d_6_, 50 MHz).
(TIF)

Figure S5 ^1^H NMR spectrum of 4c (DMSO-d_6_, 200 MHz).
(TIF)

Figure S6 ^13^C NMR spectrum of 4c (DMSO-d_6_, 50 MHz).
(TIF)

Figure S7 ^1^H NMR spectrum of 4d (DMSO-d_6_, 400 MHz).
(TIF)

Figure S8 ^13^C NMR spectrum of 4d (DMSO-d_6_, 100 MHz).
(TIF)

Figure S9 ^1^H NMR spectrum of 4e (DMSO-d_6_, 200 MHz).
(TIF)

Figure S10 ^13^C NMR spectrum of 4e (DMSO-d_6_, 50 MHz).
(TIF)

Figure S11 ^1^H NMR spectrum of 4f (DMSO-d_6_, 200 MHz).
(TIF)

Figure S12 ^1^H NMR spectrum of 4 g (DMSO-d_6_, 300 MHz, t∼40°C).
(TIF)

Figure S13 ^1^H NMR spectrum of 4 g (DMSO-d_6_, 300 MHz, t∼90°C).
(TIF)

Figure S14 1D NOESY spectrum of 4 g (DMSO-_6_, 300 MHz). Irradiation at 11.48 ppm.
(TIF)

Figure S15 1D NOESY spectrum of 4 g (DMSO-d_6_, 300 MHz). Irradiation at 8.26 ppm.
(TIF)

Figure S16 ^13^C NMR spectrum of 4 g (DMSO-d_6_, 75 MHz).
(TIF)

Figure S17 ^1^H NMR spectrum of 9 (DMSO-d_6_, 200 MHz, t∼ 40°C).
(TIF)

Figure S18 ^1^H NMR spectrum of 9 (DMSO-d_6_, 300 MHz, t∼ 90°C).
(TIF)

Figure S19 ^1^H NMR spectrum of 4 h (DMSO-d_6_, 400 MHz).
(TIF)

Figure S20 ^1^H NMR spectrum of 15 (DMSO-d_6_, 200 MHz).
(TIF)

Figure S21 ^13^C NMR spectrum of 15 (DMSO-d_6_, 50 MHz).
(TIF)

Figure S22 Chromatogram of compound 4 g obtained from reversed-phase HPLC studies.
(TIF)

Figure S23 1D NOESY spectrum of (*E*)-*N*’-benzylidene-2-(3-*tert*-butyl-1-phenyl-1*H*-pyrazol-5-ylamino)acetohydrazide (4 g) in DMSO-d_6_ (300 MHz). Irradiation at 11.48 ppm (A) and 8.26 ppm (B).
(TIF)

Table S1 p38α MAPK inhibitory activity of compounds (4a–g) at 10 µM.
(DOC)
