# Supplementary figures and images for: Discovery of Novel Orally Active Anti-Inflammatory N-Phenylpyrazolyl-N-Glycinyl-Hydrazone Derivatives That Inhibit TNF-α Production
Source: PLoS One. 2012 Oct 8;7(10):e46925. doi: 10.1371/journal.pone.0046925 (PMC3466213; doi:10.1371/journal.pone.0046925)

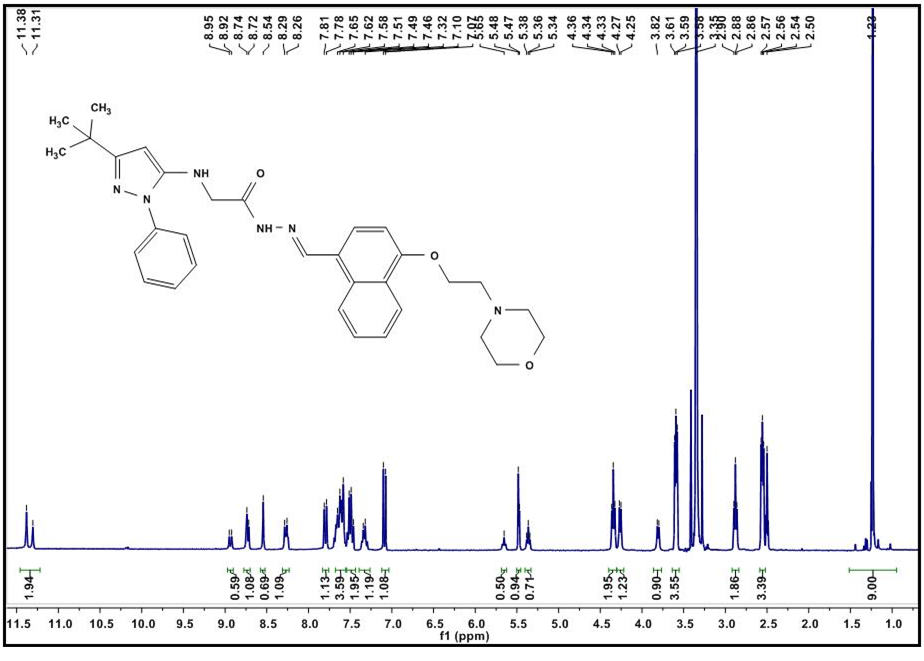

Supplement: Figure S1 — 1H NMR spectrum of 4a (DMSO-d6, 300 MHz). (TIF) [file pone.0046925.s001.tif]

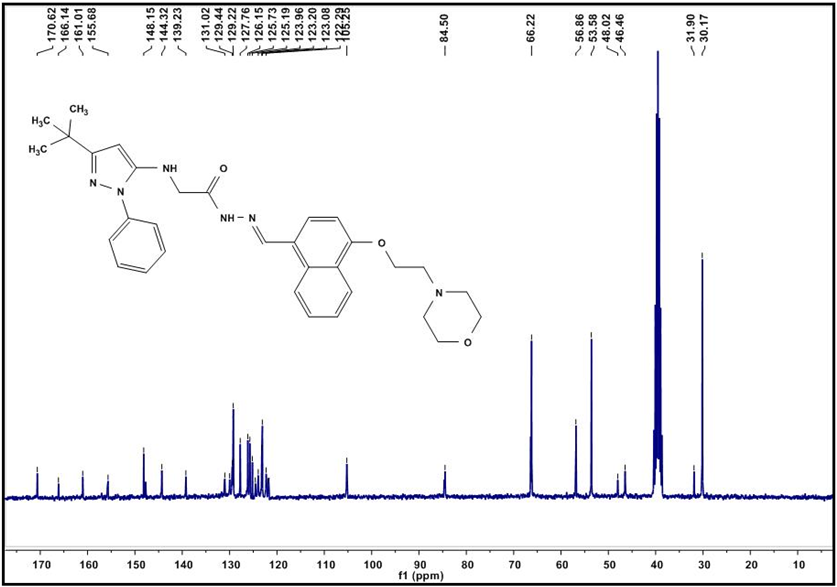

Supplement: Figure S2 — 13C NMR spectrum of 4a (DMSO-d6, 75 MHz). (TIF) [file pone.0046925.s002.tif]

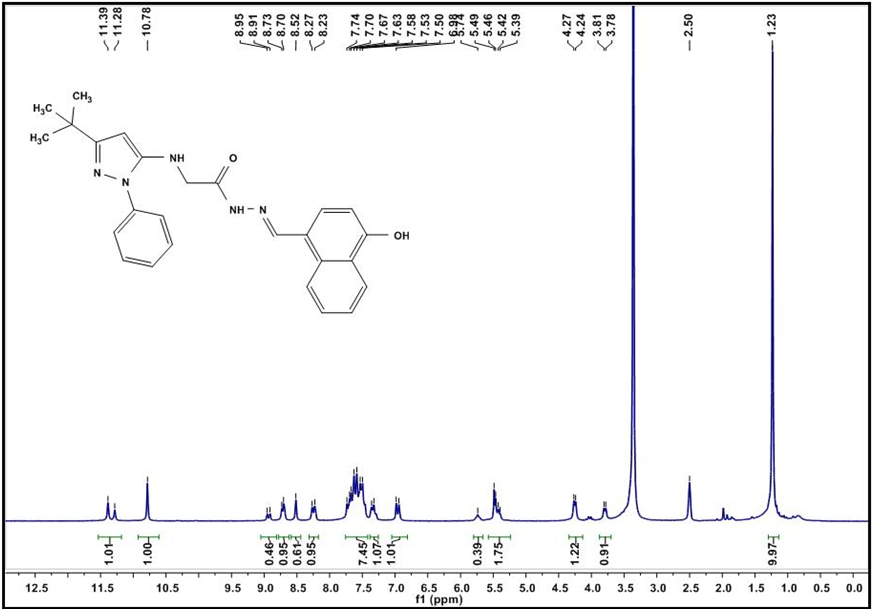

Supplement: Figure S3 — 1H NMR spectrum of 4b (DMSO-d6, 200 MHz). (TIF) [file pone.0046925.s003.tif]

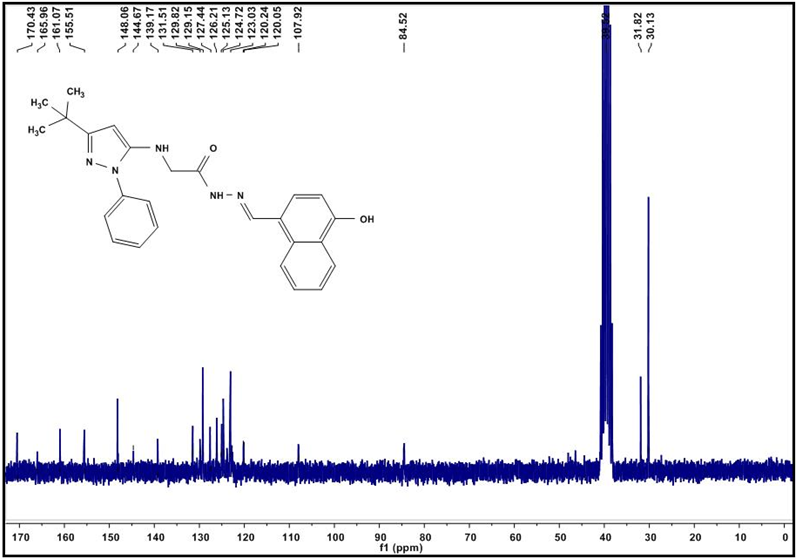

Supplement: Figure S4 — 13C NMR spectrum of 4b (DMSO-d6, 50 MHz). (TIF) [file pone.0046925.s004.tif]

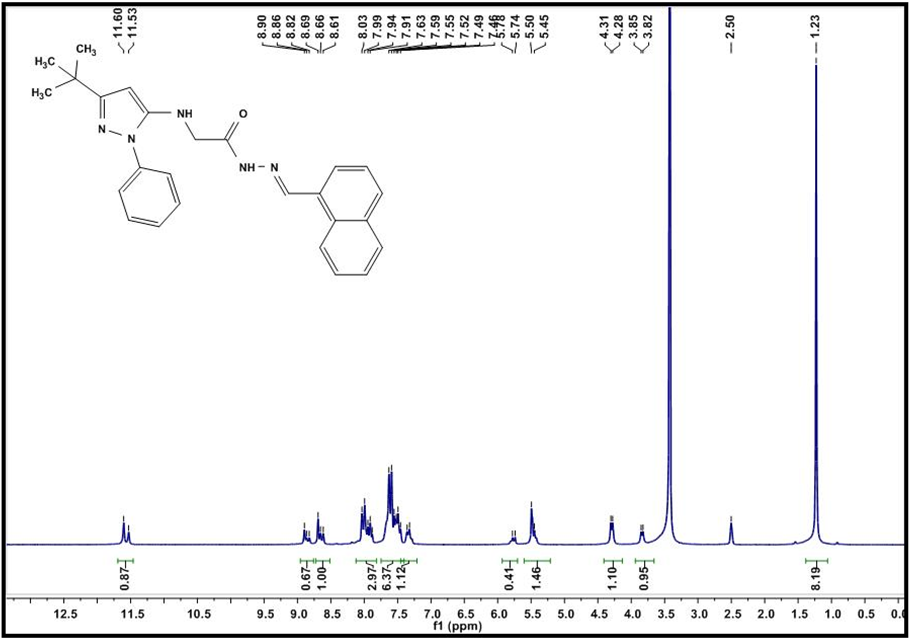

Supplement: Figure S5 — 1H NMR spectrum of 4c (DMSO-d6, 200 MHz). (TIF) [file pone.0046925.s005.tif]

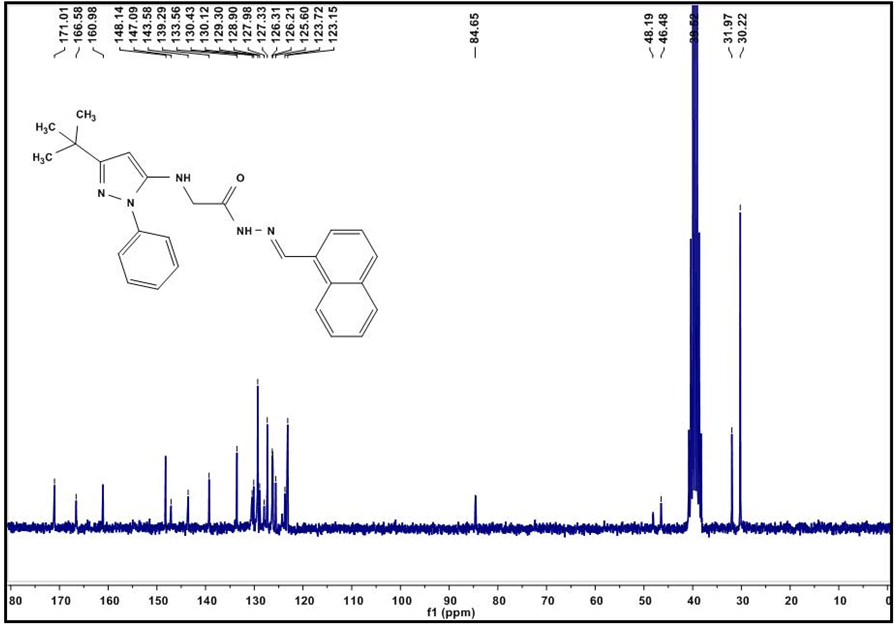

Supplement: Figure S6 — 13C NMR spectrum of 4c (DMSO-d6, 50 MHz). (TIF) [file pone.0046925.s006.tif]

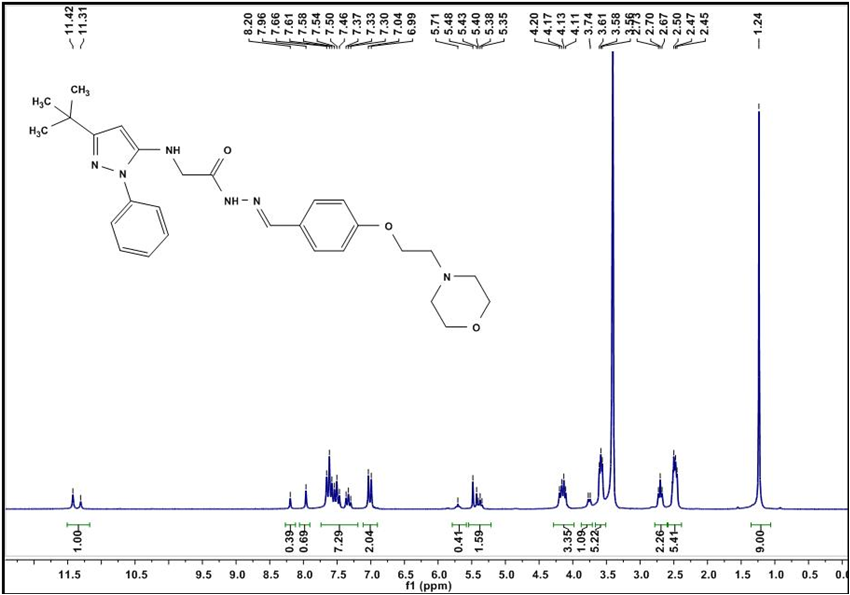

Supplement: Figure S7 — 1H NMR spectrum of 4d (DMSO-d6, 400 MHz). (TIF) [file pone.0046925.s007.tif]

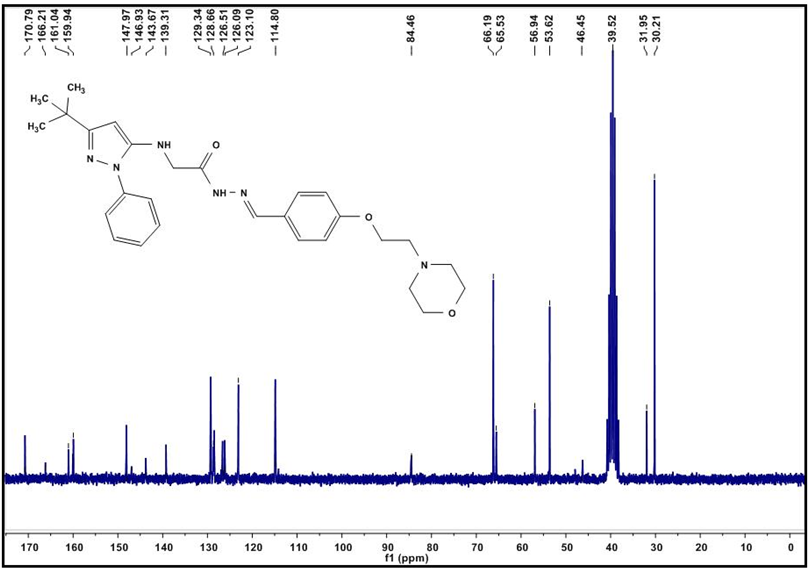

Supplement: Figure S8 — 13C NMR spectrum of 4d (DMSO-d6, 100 MHz). (TIF) [file pone.0046925.s008.tif]

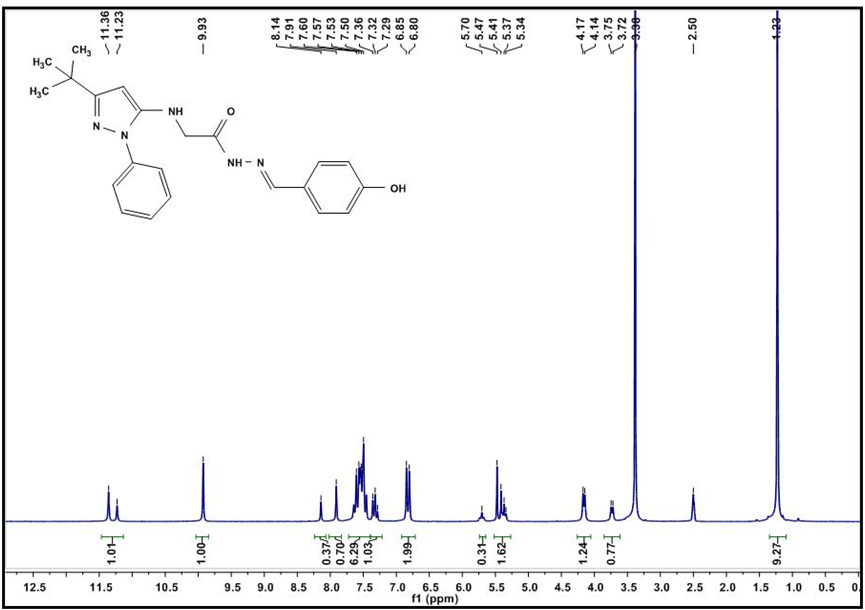

Supplement: Figure S9 — 1H NMR spectrum of 4e (DMSO-d6, 200 MHz). (TIF) [file pone.0046925.s009.tif]

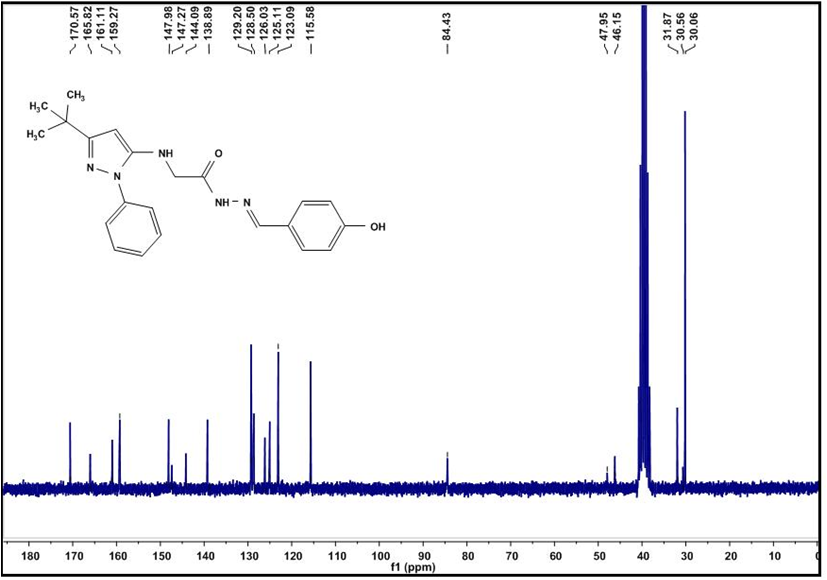

Supplement: Figure S10 — 13C NMR spectrum of 4e (DMSO-d6, 50 MHz). (TIF) [file pone.0046925.s010.tif]

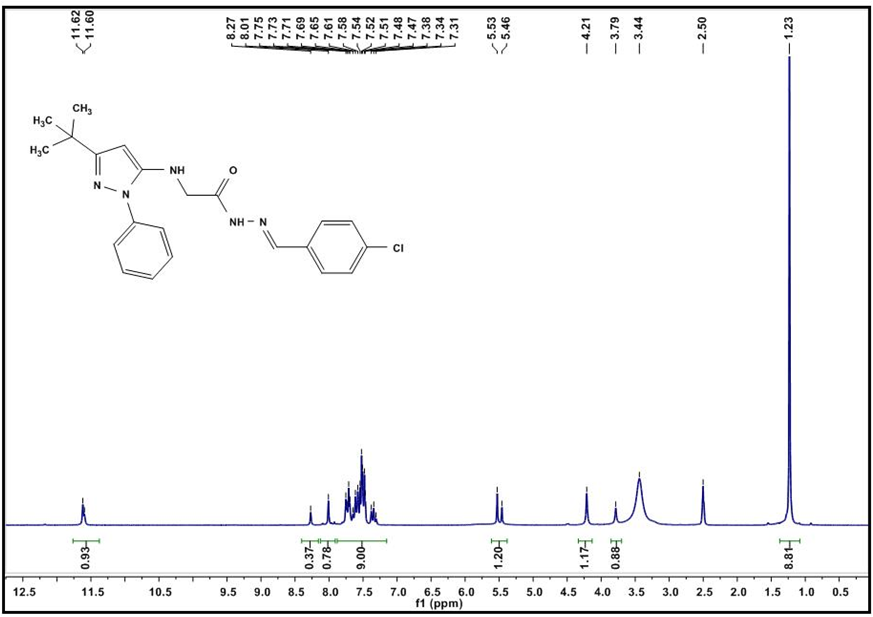

Supplement: Figure S11 — 1H NMR spectrum of 4f (DMSO-d6, 200 MHz). (TIF) [file pone.0046925.s011.tif]

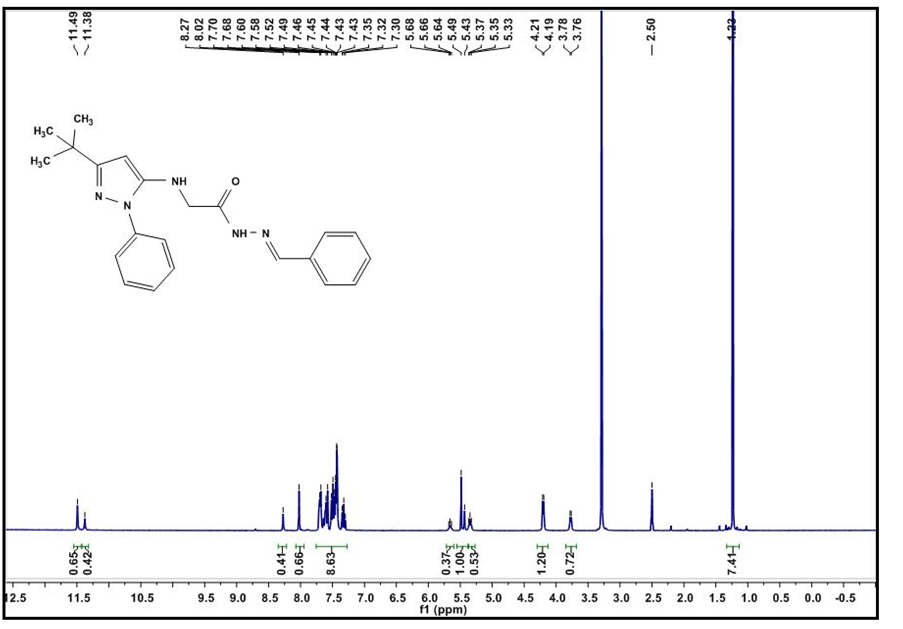

Supplement: Figure S12 — 1H NMR spectrum of 4 g (DMSO-d6, 300 MHz, t∼40°C). (TIF) [file pone.0046925.s012.tif]

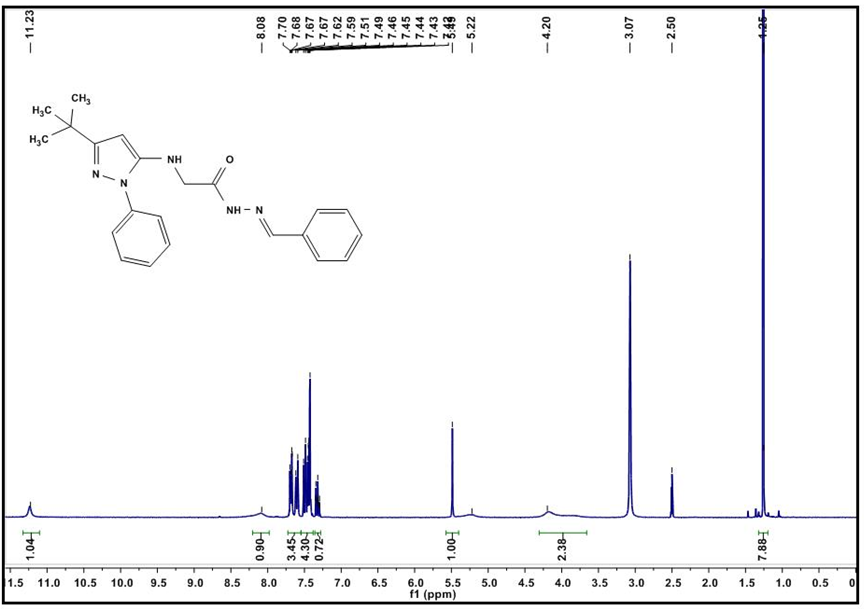

Supplement: Figure S13 — 1H NMR spectrum of 4 g (DMSO-d6, 300 MHz, t∼90°C). (TIF) [file pone.0046925.s013.tif]

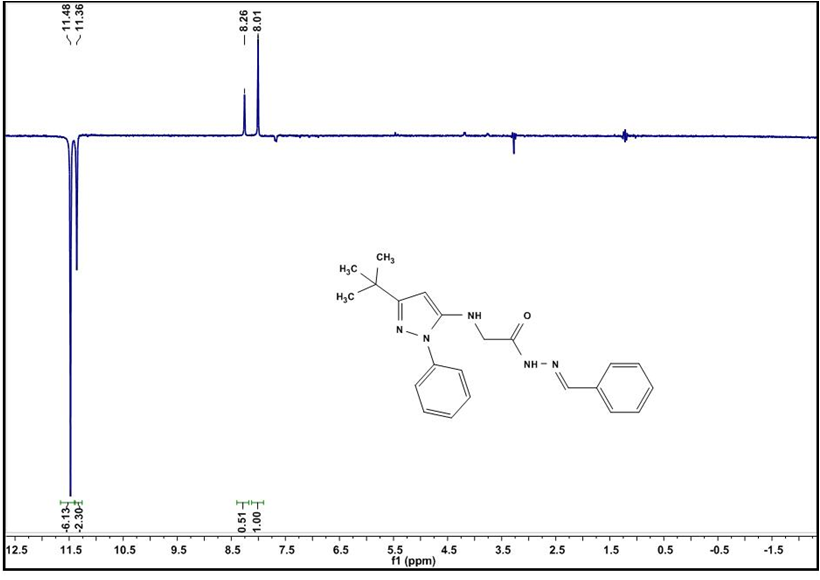

Supplement: Figure S14 — 1D NOESY spectrum of 4 g (DMSO-6, 300 MHz). Irradiation at 11.48 ppm. (TIF) [file pone.0046925.s014.tif]

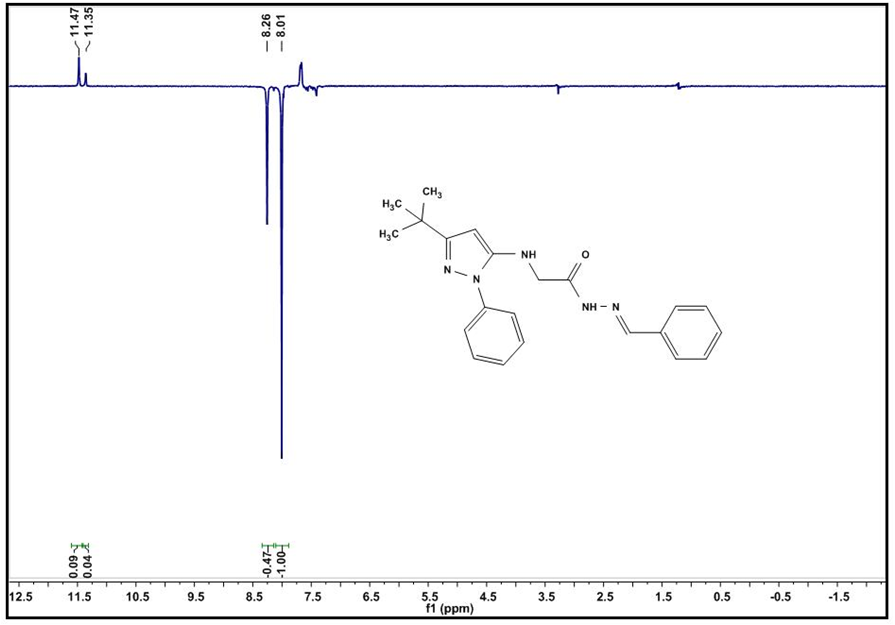

Supplement: Figure S15 — 1D NOESY spectrum of 4 g (DMSO-d6, 300 MHz). Irradiation at 8.26 ppm. (TIF) [file pone.0046925.s015.tif]

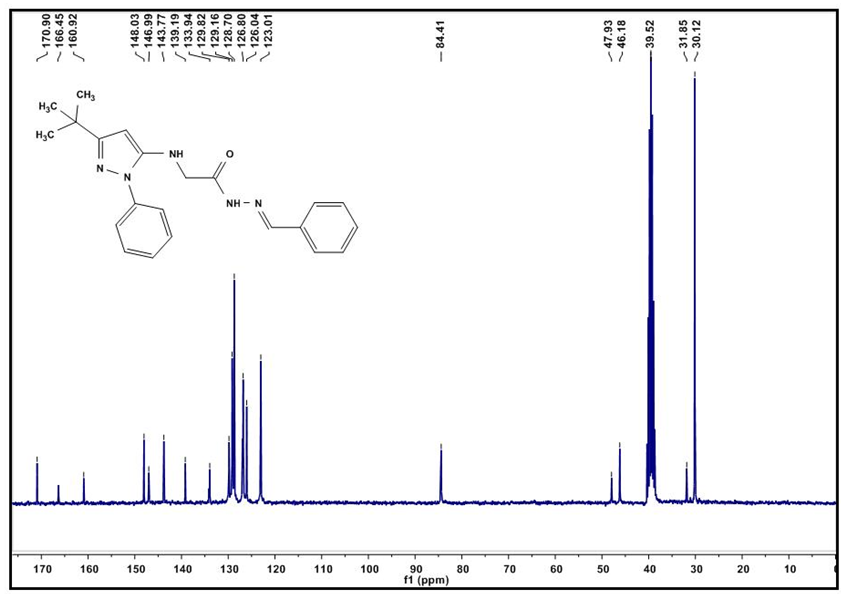

Supplement: Figure S16 — 13C NMR spectrum of 4 g (DMSO-d6, 75 MHz). (TIF) [file pone.0046925.s016.tif]

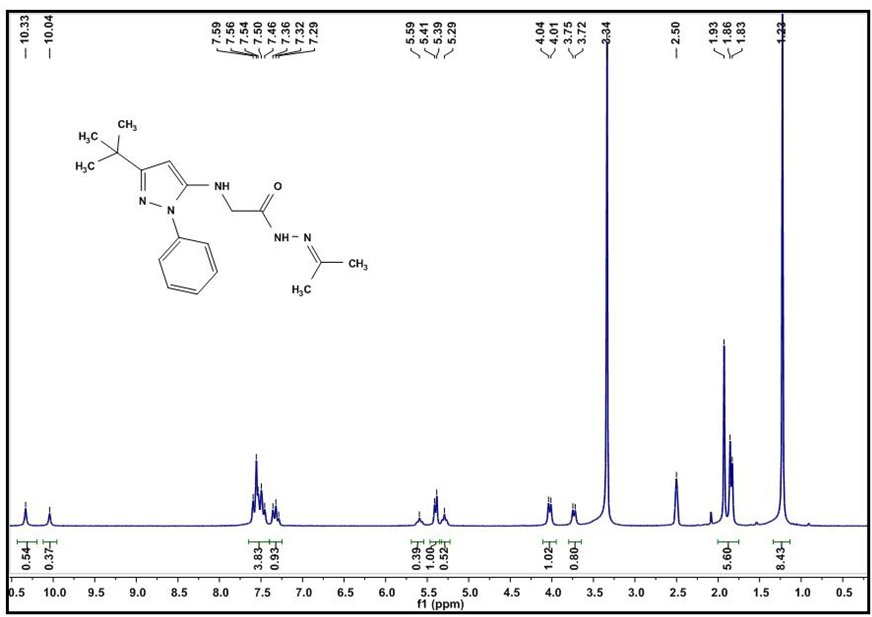

Supplement: Figure S17 — 1H NMR spectrum of 9 (DMSO-d6, 200 MHz, t∼ 40°C). (TIF) [file pone.0046925.s017.tif]

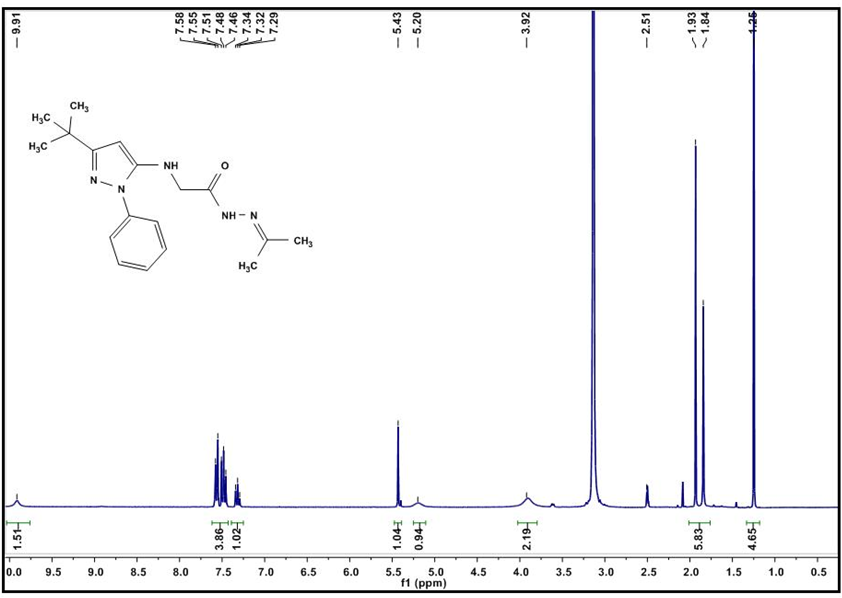

Supplement: Figure S18 — 1H NMR spectrum of 9 (DMSO-d6, 300 MHz, t∼ 90°C). (TIF) [file pone.0046925.s018.tif]

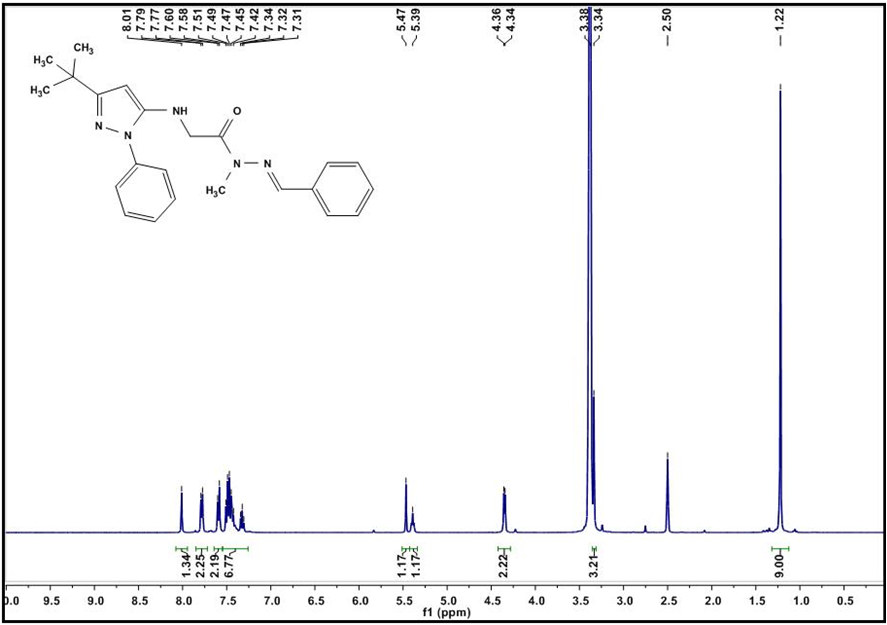

Supplement: Figure S19 — 1H NMR spectrum of 4 h (DMSO-d6, 400 MHz). (TIF) [file pone.0046925.s019.tif]

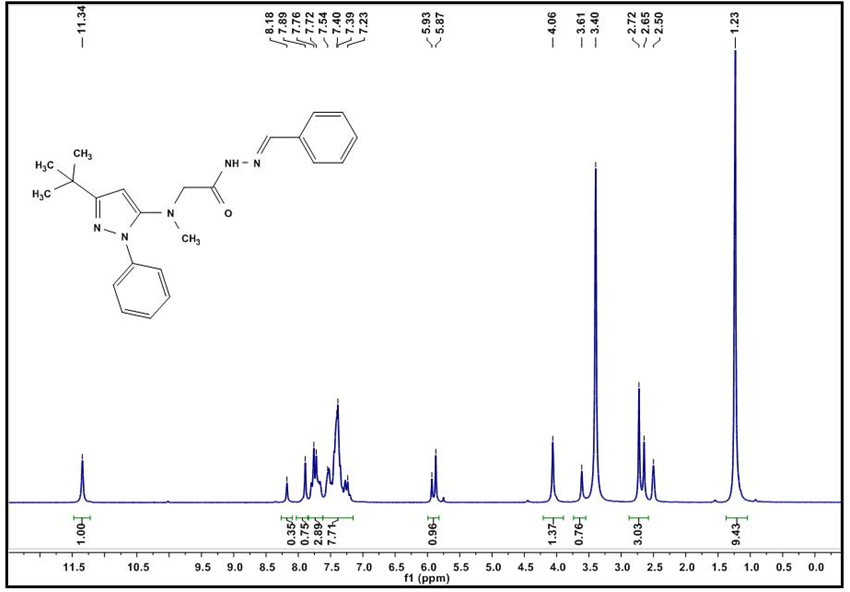

Supplement: Figure S20 — 1H NMR spectrum of 15 (DMSO-d6, 200 MHz). (TIF) [file pone.0046925.s020.tif]

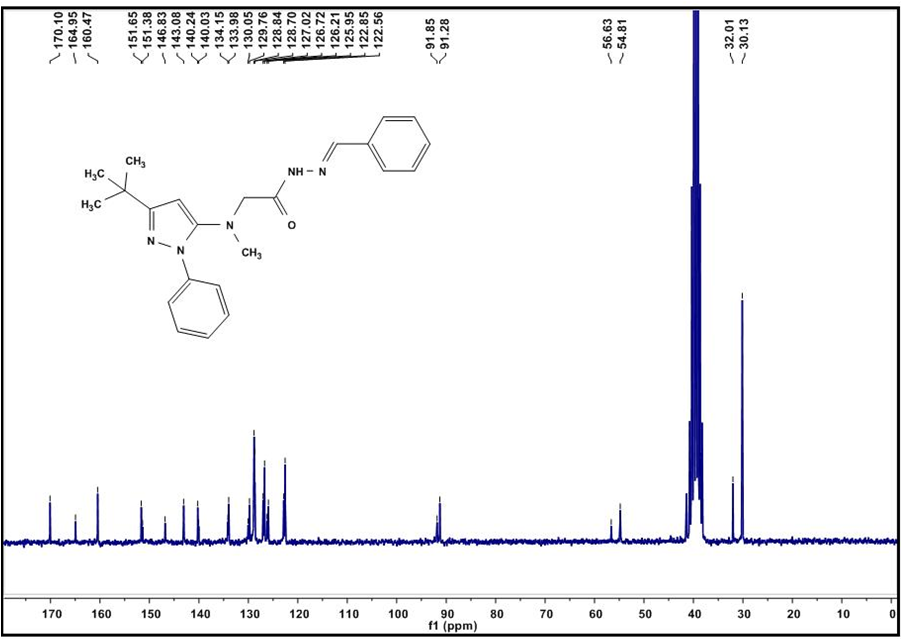

Supplement: Figure S21 — 13C NMR spectrum of 15 (DMSO-d6, 50 MHz). (TIF) [file pone.0046925.s021.tif]

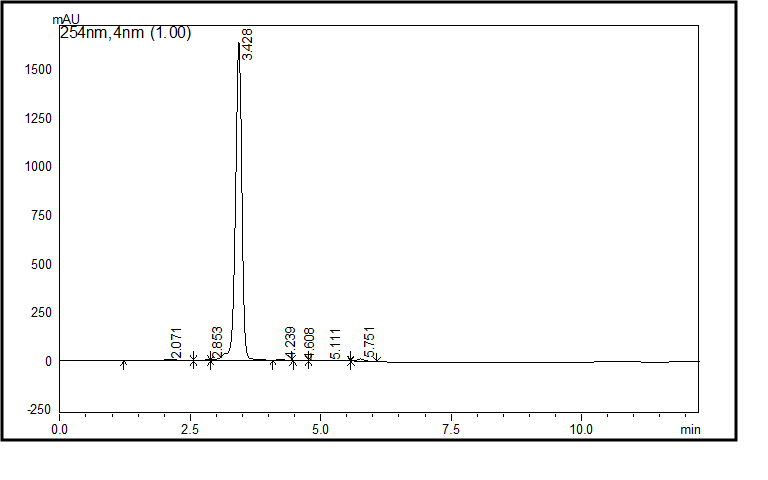

Supplement: Figure S22 — Chromatogram of compound 4 g obtained from reversed-phase HPLC studies. (TIF) [file pone.0046925.s022.tif]

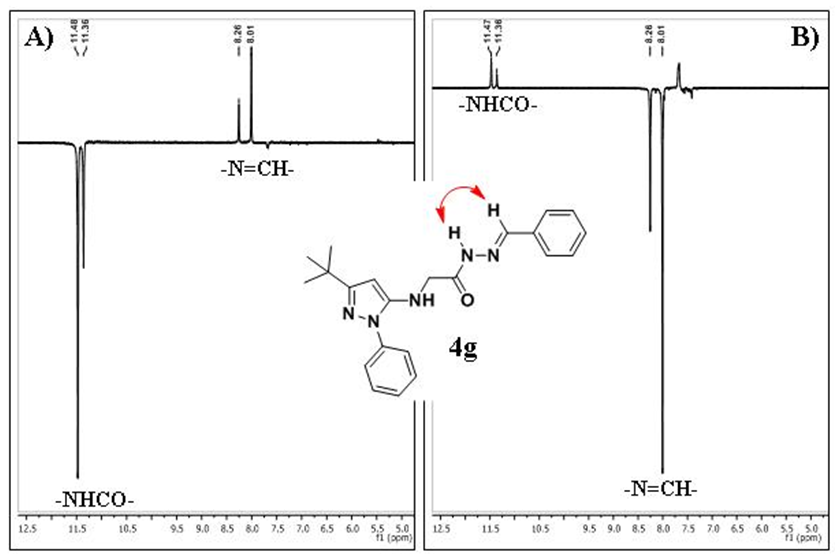

Supplement: Figure S23 — 1D NOESY spectrum of (E)-N’-benzylidene-2-(3-tert-butyl-1-phenyl-1H-pyrazol-5-ylamino)acetohydrazide (4 g) in DMSO-d6 (300 MHz). Irradiation at 11.48 ppm (A) and 8.26 ppm (B). (TIF) [file pone.0046925.s023.tif]
