# Supplementary material for: Discovery of Novel Orally Active Anti-Inflammatory N-Phenylpyrazolyl-N-Glycinyl-Hydrazone Derivatives That Inhibit TNF-α Production
Source: PLoS One. 2012 Oct 8;7(10):e46925. doi: 10.1371/journal.pone.0046925 (PMC3466213; doi:10.1371/journal.pone.0046925)
Supplement: Table S1 — p38α MAPK inhibitory activity of compounds (4a–g) at 10 µM. (DOC) [file pone.0046925.s024.doc]

**Table S1.** p38α MAPK inhibitory activity of compounds (4a-g) at 10 μM.

| **Compound** | **% inhibition of p38α MAPK*** |
| --- | --- |
| 4a | -5,35 |
| 4b | 33,26 |
| 4c | 19,71 |
| 4d | 16,40 |
| 4e | 31,78 |
| 4f | 15,29 |
| 4g | 19,46 |

*This assay was performed as described in reference 23.
